# Genetic regulation of l-tryptophan metabolism in *Psilocybe mexicana* supports psilocybin biosynthesis

**DOI:** 10.1186/s40694-024-00173-6

**Published:** 2024-04-25

**Authors:** Paula Sophie Seibold, Sebastian Dörner, Janis Fricke, Tim Schäfer, Christine Beemelmanns, Dirk Hoffmeister

**Affiliations:** 1 Institute for Pharmacy, Friedrich Schiller University Jena, Winzerlaer Strasse 2, 07745 Jena, Germany; 2 Pharmaceutical Microbiology, Leibniz Institute for Natural Product Research and Infection Biology – Hans Knöll Institute, Beutenbergstr. 11a, 07745 Jena, Germany; 3 Cluster of Excellence Balance of the Microverse, Friedrich Schiller University Jena, Neugasse 23, 07743 Jena, Germany; 4 Chemical Biology of Microbe-Host Interactions, Leibniz Institute for Natural Product Research and Infection Biology – Hans Knöll Institute, Beutenbergstr. 11a, 07745 Jena, Germany; 5 Helmholtz Institute for Pharmaceutical Research Saarland (HIPS), Helmholtz Centre for Infection Research (HZI), Campus E8.1, 66123 Saarbrücken, Germany; 6 Saarland University, 66123 Saarbrücken, Germany

**Keywords:** Aromatic acetaldehyde synthase, Basidiomycota, Metabolic flux, Psilocybin, Tryptophan

## Abstract

**Background:**

Although Basidiomycota produce pharmaceutically and ecologically relevant natural products, knowledge of how they coordinate their primary and secondary metabolism is virtually non-existent. Upon transition from vegetative mycelium to carpophore formation, mushrooms of the genus *Psilocybe* use l-tryptophan to supply the biosynthesis of the psychedelic tryptamine alkaloid psilocybin with the scaffold, leading to a strongly increased demand for this particular amino acid as this alkaloid may account for up to 2% of the dry mass. Using *Psilocybe mexicana* as our model and relying on genetic, transcriptomic, and biochemical methods, this study investigated if l-tryptophan biosynthesis and degradation in *P. mexicana* correlate with natural product formation.

**Results:**

A comparative transcriptomic approach of gene expression in *P. mexicana* psilocybin non-producing vegetative mycelium versus producing carpophores identified the upregulation of l-tryptophan biosynthesis genes. The shikimate pathway genes *trpE1*, *trpD*, and *trpB* (encoding anthranilate synthase, anthranilate phosphoribosyltransferase, and l-tryptophan synthase, respectively) were upregulated in carpophores. In contrast, genes *idoA* and *iasA*, encoding indole-2,3-dioxygenase and indole-3-acetaldehyde synthase, i.e., gateway enzymes for l-tryptophan-consuming pathways, were massively downregulated. Subsequently, IasA was heterologously produced in *Escherichia coli* and biochemically characterized in vitro. This enzyme represents the first characterized microbial l-tryptophan-preferring acetaldehyde synthase. A comparison of transcriptomic data collected in this study with prior data of *Psilocybe cubensis* showed species-specific differences in how l-tryptophan metabolism genes are regulated, despite the close taxonomic relationship.

**Conclusions:**

The upregulated l-tryptophan biosynthesis genes and, oppositely, the concomitant downregulated genes encoding l-tryptophan-consuming enzymes reflect a well-adjusted cellular system to route this amino acid toward psilocybin production. Our study has pilot character beyond the genus *Psilocybe* and provides, for the first time, insight in the coordination of mushroom primary and secondary metabolism.

**Supplementary Information:**

The online version contains supplementary material available at 10.1186/s40694-024-00173-6.

## Introduction

The Basidiomycota have collectively evolved a prolific specialized, so-called secondary metabolism. These pathways elaborate a rich and structurally diverse repertoire of bioactive natural products, among them toxicologically, pharmaceutically or ecologically relevant molecules [1]. Ubiquitous compounds of the central or primary metabolism, such as acetyl-CoA or amino acids, serve as precursors to supply the main building blocks to the biosynthesis pathways [2, 3]. Generally, primary meta-bolism uses salvage pathways to regenerate metabolites whereas secondary metabolism culminates in accumulated or secreted end products. Therefore, upon eliciting natural product pathways, the demand for the precursors increases massively which implies a well-adjusted interplay between primary and secondary metabolism. However, knowledge of how basidiomycetes coordinate their primary and secondary metabolism is very limited.

**Fig. 1:**
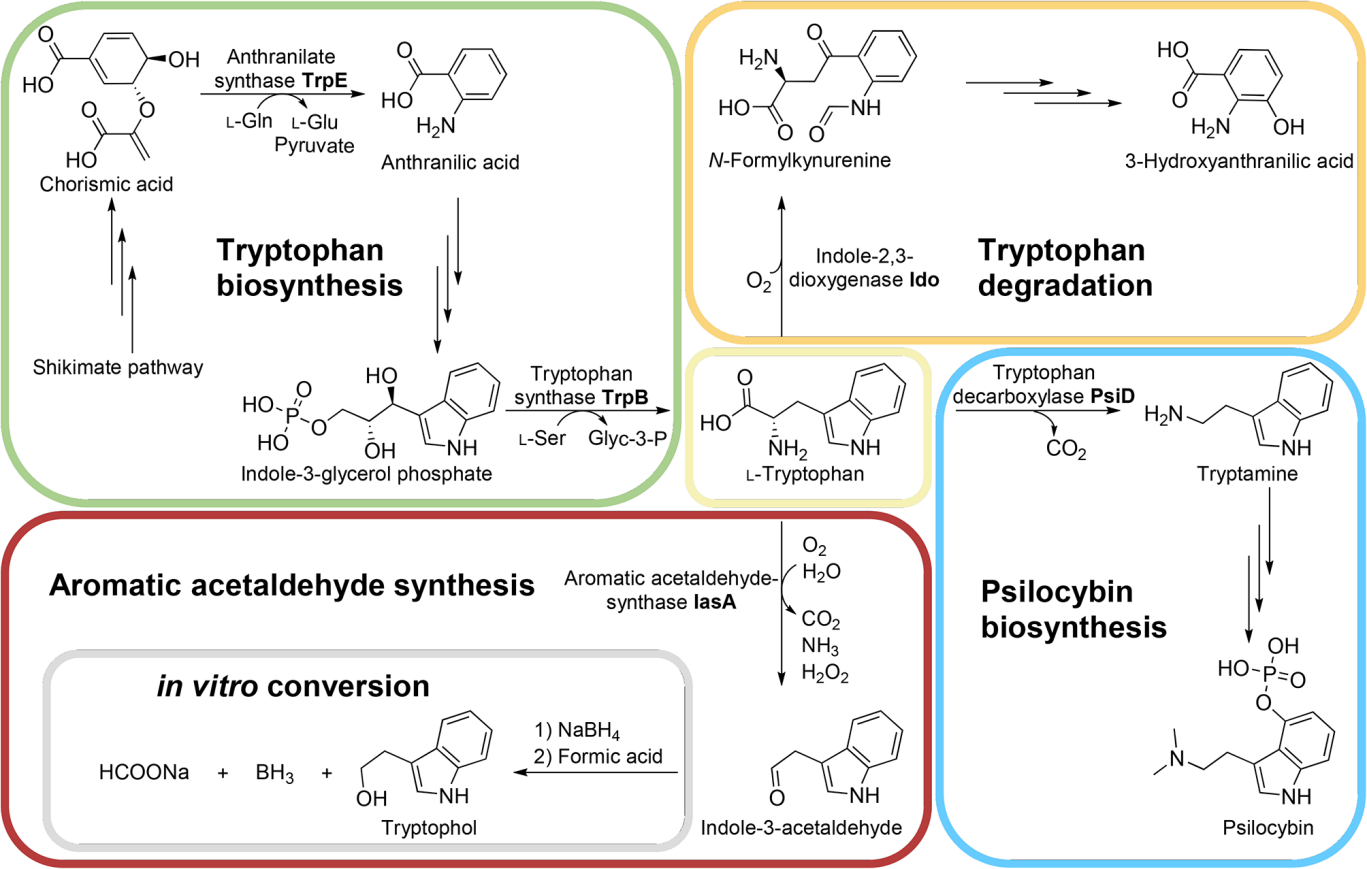
Selected pathways and enzymes of the tryptophan metabolism in *P. mexicana*. Tryptophan catabolism occurs via the kynurenine pathway, psilocybin biosynthesis and aromatic acetaldehyde synthesis. Indole-3-acetaldehyde was reduced to tryptophol in vitro by adding NaBH_4_

Mushrooms of the basidiomycete genus *Psilocybe*, notorious for its perception-altering effects [4–6], produce psilocybin which serves as prodrug for psilocin, the psychotropic and chemically reactive dephosphorylated follow-up compound (Fig. 1). Psilocybin biosynthesis is initiated by l-tryptophan decarboxylation, mediated by the decarboxylase PsiD [7]. The activity of this metabolic pathway depends on the developmental stage and increases strongly upon fructification that, in return, is triggered by light [8, 9]. Consequently, during carpophore formation, the demand for l-tryptophan increases drastically, given that psilocybin accounts for up to 2% of the mushroom dry mass [10–15]. In *P. cubensis*, the *psiD* gene is 395-fold upregulated when mushroom primordia are formed [7, 8]. However, the adjustment of metabolic pathways supplying or degrading l-tryptophan is unknown and it has remained shrouded how the fungus meets the demand when psilocybin production sets in.

Aromatic l-amino acids are biosynthesized by the shikimate pathway [16]. From the intermediate chorismate, the anabolism of l-tryptophan branches off by anthranilate synthesis, catalyzed by TrpE (Fig. 1 and Additional file 1: Figure S1). Three further reactions ultimately lead to the formation of l-tryptophan to supply protein biosynthesis and other pathways that require tryptophan and that represent tryptophan sinks, besides psilocybin assembly. For example, indole-2,3-dioxygenases (IDOs) initiate the pathway to 3-hydroxyanthranilate via kynurenine as the starting point for nicotinamide metabolism [17]. Likewise, indole acetaldehyde synthase depends on l-tryptophan supply (Fig. 1). In this study, we present a transcriptomic analysis of *P. mexicana* with particular emphasis on genes involved in the l-tryptophan meta-bolism. We investigated how the genes of the tryptophan branch of the shikimate pathway are regulated along with genes encoding IDOs as well as an indole-3-acetaldehyde synthase. The latter was recombinantly produced and biochemically characterized to verify its activity, given that microbial indole-3-acetaldehyde synthases have not been investigated yet.

## Results

### Transcriptomic analysis of *P. mexicana*

For insight into the regulation of tryptophan biosynthetic genes, a transcriptomic study was performed. First, we needed to design a robust experimental set-up to compare psilocybin-producing and non-producing conditions. Previous investigations of dried *P. mexicana* sclerotia and carpophores determined psilocybin contents up to 0.65% and 0.39%, respectively [13, 18]. Prior efforts to optimize media usually aimed at increased psilocybin concentrations [19]. We systematically tested various media and found FB3G medium suitable for comparison as vegetative mycelium grown in this medium was virtually free of psilocybin whereas BNM medium stimulated psilocybin production (Additional file 1: Figures S2 and S3, media composition described in methods section) [19]. Consequently, comparative RNA-Seq was performed with RNA samples isolated from vegetative mycelium, grown either in FB3G or BNM medium, and from *P. mexicana* carpophores. Overall, 289,463,012 reads yielding over 86 Gb of sequence data were obtained with a mean quality score of 35.57. Details of the DESeq2 analysis are shown in Additional file 1: Figures S4-S10, the numbers of up- and downregulated genes (threshold criteria: log_2_-fold change > │1│ and adjusted *p*-value (*p*_adj_) < 0.05) are provided in Additional file 1: Table S1.

### Differential expression of genes for l-tryptophan anabolism

**Fig. 2:**
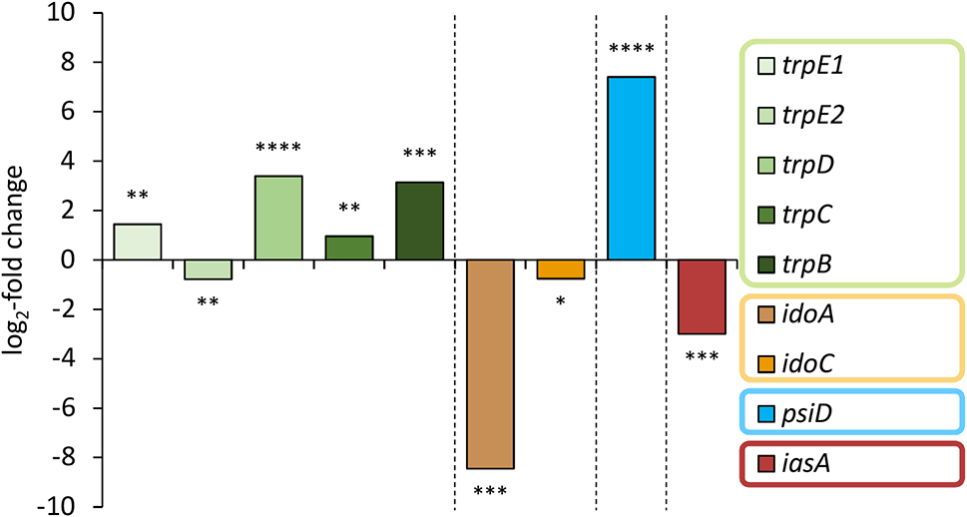
Expression analysis of selected genes involved in the tryptophan metabolism in *P. mexicana* based on RNA-Seq data. DESeq2 analysis compared mycelium submerse-grown in FB3G versus carpophores. Genes that are upregulated in carpophores versus submerse-grown mycelium in FB3G show positive log_2_-fold changes. Asterisks represent the calculated adjusted *p*-values: * 0.05 < *p*_adj_; ** 1∙10^− 10^ < *p*_adj_ ≤ 0.05; ** 1∙10^− 100^ < *p*_adj_ ≤ 1∙10^− 10^; **** *p*_adj_ ≤ 1∙10^− 100^. Color coding: green – tryptophan biosynthesis, orange/brown – tryptophan degradation, blue – psilocybin biosynthesis, maroon – aromatic acetaldehyde synthesis

We first investigated genes implicated in tryptophan anabolism, a generally well understood process in model organisms such as yeast and Aspergilli [20, 21]. The conversion of chorismate to anthranilate and further to l-tryptophan is catalyzed by the combined action of four mono- or multifunctional enzymes that form a branch of the shikimate pathway (Additional file 1: Figure S1). These include (i) anthranilate synthase TrpE as the first enzyme of the branch, (ii) anthranilate phosphoribosyltransferase TrpD, (iii) TrpC, a tri-functional enzyme providing glutamine amidotransferase (G domain), phosphoribosyl anthranilate isomerase (F domain) and indole-3-glycerol phosphate synthase activity (C domain), and finally (iv) the homodimeric tryptophan synthase TrpB featuring an α- and a β-domain per monomer [22]. Prior to investigating the transcriptional dynamics, the respective genes needed to be identified in the genome of *P. mexicana*. Therefore, BLAST analyses were performed with annotated fungal tryptophan pathway genes [23] (Additional file 1: Table S2). In fact, pronounced transcriptional changes were found when comparing the data of FB3G mycelium (psilocybin biosynthesis suppressed) with the carpophore samples (psilocybin biosynthesis induced, Fig. 2, Additional file 1: Figure S11 and Table S3) for the expression of the genes putatively encoding TrpE, TrpD and TrpB. These were strongly upregulated in carpophores (*trpE1*: 2.7-fold; *trpD*: 10.5-fold; *trpB*: 8.8-fold, corresponding log_2_-fold values are 1.45, 3.39 and 3.14). A gene putatively encoding a second anthranilate synthase, TrpE2, was only minimally downregulated (1.7-fold) which may reflect the frequently observed phenomenon of multiple (yet possibly non-functional) alleles of biosynthetic genes encoded in basidiomycete genomes [24–26]. With a 1.9-fold upregulation, the transcriptional activity of the *trpC* gene changed at a lower degree. Still, the more strongly upregulated tryptophan biosynthesis genes *trpE1, trpD* and *trpB* are consistent with the increasing demand for l-tryptophan in carpophores when psilocybin biosynthesis sets in.

### Differential expression of genes for l-tryptophan-converting enzymes

**Fig. 3:**
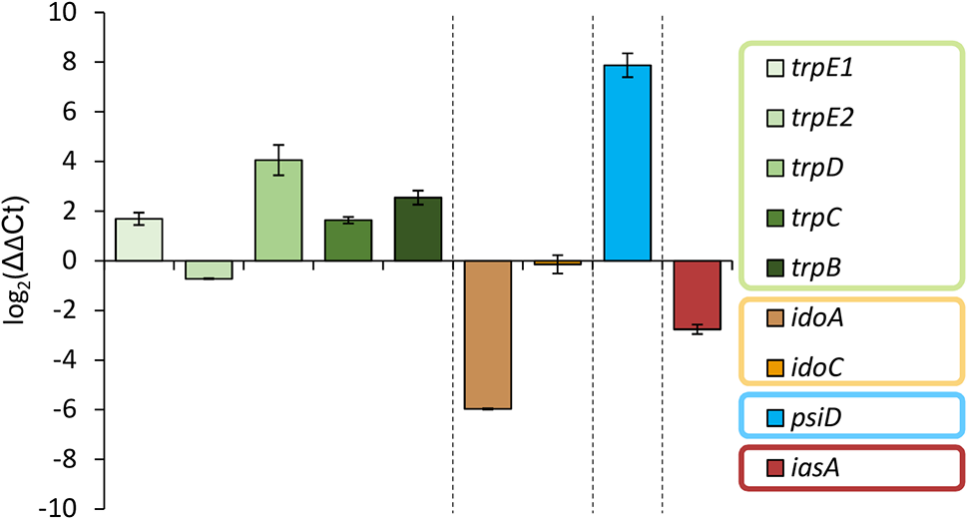
Expression analysis of selected genes involved in the tryptophan metabolism in *P. mexicana* based on qRT-PCR results. The analysis compared mycelium submerse-grown in FB3G medium and carpophores. Shown values represent log_2_-fold changes (positive, if genes are upregulated in carpophores) and standard deviations of means (*n* = 3). The values are normalized to the expression of *enoA* (encoding enolase) as a control gene. Color coding: green – tryptophan biosynthesis, orange/brown – tryptophan degradation, blue – psilocybin biosynthesis, maroon – aromatic acetaldehyde synthesis

Subsequently, we analyzed the genes encoding key enzymes that convert l-tryptophan (Fig. 1). Aromatic acetaldehyde synthases (AASs) draw on the l-tryptophan pool by producing indole-3-acetaldehyde in a single combined decarboxylation/deamination step. Likewise, indoleamine-2,3-dioxygenases (IDOs) degrade l-tryptophan as they catalyze the oxidative cleavage of the pyrrol ring to yield *N*-formylkynurenine, thereby supplying various pathways with substrate, among them one leading to 3-hydroxyanthranilic acid and nicotinamide/NAD^+^. In fact, the expression of putative genes for an acetaldehyde synthase (IasA) as well as for IDOs was downregulated in mushrooms (*iasA*: eight-fold; *idoA*: 350-fold; *idoC*: 1.7-fold). The corresponding log_2_-fold changes are − 3.0, -8.45, and − 0.76, respectively (Additional file 1: Table S3). A pathway-specific l-tryptophan decarboxylase is the gateway enzyme of the psilocybin biosynthesis [7] and, thus, represents an l-tryptophan sink as well. In contrast to the downregulated genes for IDOs and IasA, the *psiD* gene encoding this decarboxylase [27], was 170-fold upregulated in carpophores. The latter value confirms previous findings for *P. cubensis psiD* that is massively expressed in primordia and carpophores as well [8]. To confirm the RNA-Seq data, expression of these genes was independently investigated by qRT-PCR that yielded perfectly congruent results (Fig. 3). Collectively, these findings further support the notion that l-tryptophan-related genes are regulated in a fashion to supply PsiD with a maximum quantity of this aromatic amino acid upon beginning psilocybin production in carpophores. Generally, the comparison between the three conditions (carpophores, and mycelium grown in BNM and FB3G media (Additional file 1: Table S3, Figure S11)) also underlines and confirms the relevance of medium composition and developmental stage for psilocybin content.

### Characterization of *P. mexicana* IasA

**Fig. 4:**
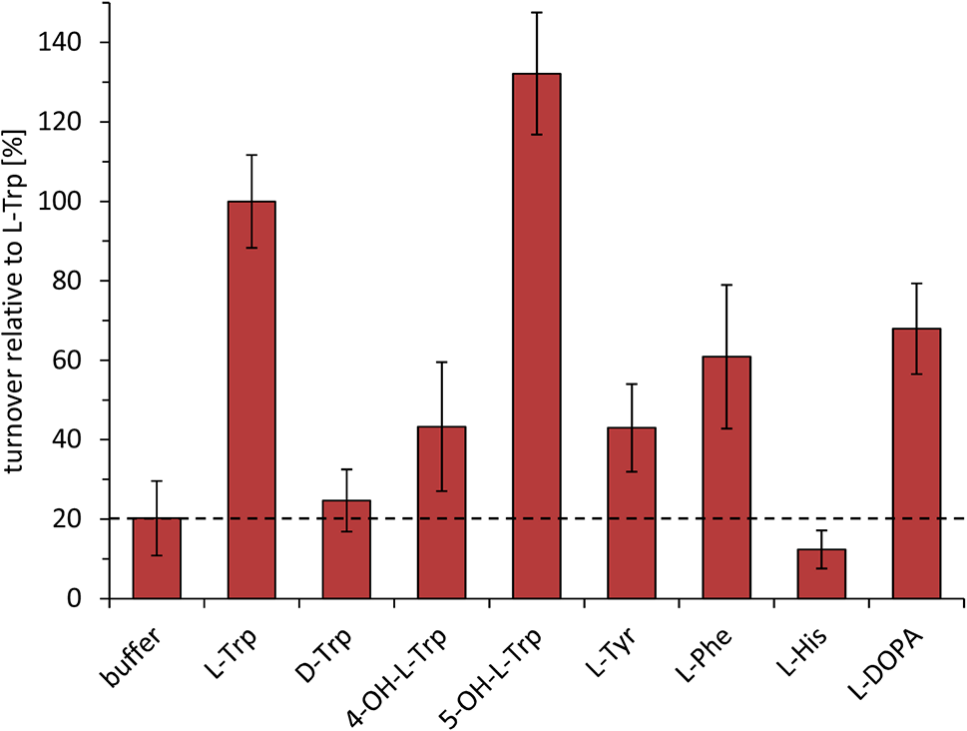
Substrate specificity of *P. mexicana* IasA. Photometric detection of hydrazone formation from IasA-produced aldehydes and 2,4-dinitrophenylhydrazine (2,4-DNPH). Absorption was measured at λ = 500 nm and 800 nm (reference wavelength). The value of the heat-inactivated control thus obtained was subtracted from the respective value of the reactions with native enzyme. The experiment was performed with two biological replicates and three technical replicates each. Mean values and standard deviations are shown

**Fig. 5:**
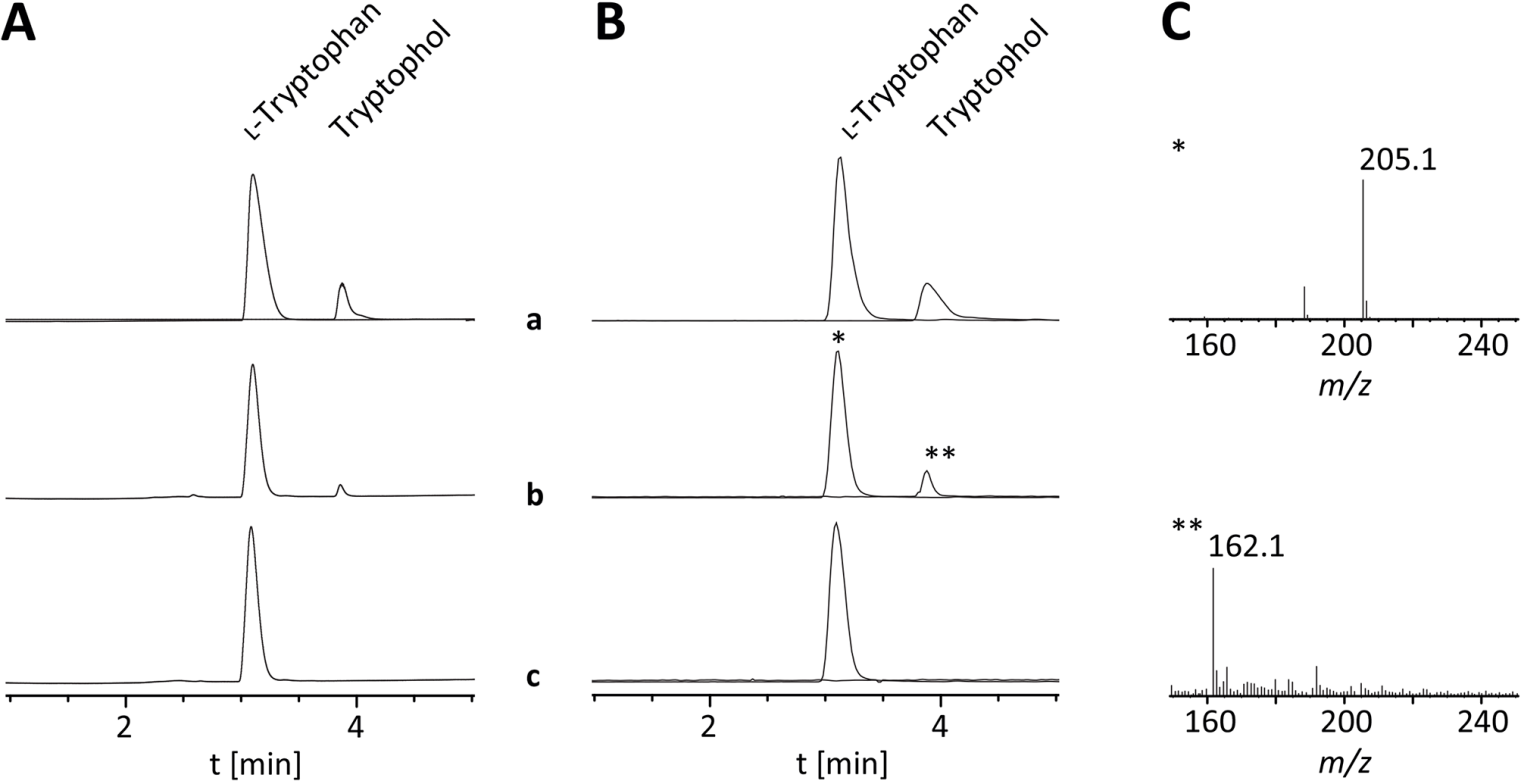
Chromatographic analysis of IasA activity assays to detect tryptophol formation by *P. mexicana* IasA. (A) Chromatograms were extracted at λ = 280 nm. Top trace a: overlaid chromatograms of l-tryptophan and tryptophol references, center trace b: reaction with IasA, bottom trace c: negative control with heat-inactivated IasA. (B) Extracted ion chromatograms (EICs; *m/z* 162 and 205 [M + H]^+^). (C) Mass spectra of chromatographic signals of l-tryptophan (*) and tryptophol (**) in trace b, recorded in positive mode

Aromatic aldehyde synthases (AASs) and aromatic amino acid decarboxylases (AAADs) share common ancestry and, consequently, very similar amino acid sequences. The decision between the two catalytic activities (decarboxylation and oxidative deamination by AASs versus decarboxylation by AAADs) is primarily mediated by one signature amino acid residue located in the large loop close to the active site (phenylalanine for AAS, tyrosine for AAADs) [28–30]. The amino acid sequence alignment of *P. mexicana* IasA with previously described AASs and AAADs identified a phenylalanine residue at position 329, which points to a function as acetaldehyde synthase (Additional file 1: Figure S12). To confirm the catalytic activity, IasA was heterologously produced and assayed in vitro. The enzyme is encoded by a 2064 bp gene, which is interrupted by ten introns between 50 and 62 bp in length. The fully spliced *iasA* reading frame is 1503 bp long and encodes a 500 aa protein with a predicted mass of 55.9 kDa. The amino acid sequence of *P. mexicana* IasA is 80% identical and 85% similar to that of *P. cubensis *l-3,4-dihydroxyphenylacetaldehyde synthase PcDHPAAS (AYU58583) (Additional file 1: Table S4). To produce recombinant enzyme, the *P. mexicana iasA* cDNA was cloned to create expression plasmid pPS66, which was used to transform *E. coli* KRX. IasA was produced as a 56.9 kDa C-terminally tagged hexahistidine fusion protein (Additional file 1: Figure S13) and purified by metal affinity chromatography. Size exclusion chromatography with urea-denatured IasA resulted in a single symmetrical peak at an elution volume of 13.4 mL (Additional file 1: Figure S14), which is consistent with the calculated monomeric mass (56.9 kDa). When native protein was loaded, IasA eluted as a single peak at 14.4 mL, corresponding to the size of a homodimer (Additional file 1: Figure S14). This result is consistent with previously described homodimeric AAADs and AASs [30]. When the *in silico* modeled structure of *P. mexicana* IasA was superimposed with the experimentally determined protein structure of *Arabidopsis thaliana* phenylacetaldehyde synthase (PDBe 6eei [30]), a high degree of structural similarity was found (Additional file 1: Figure S15). Subsequently, the enzymatic activity of IasA was assayed in PLP-containing sodium phosphate buffer (pH 7.5) and the product detected with Brady’s reagent [31]. Substrates tested included l- and d-configured tryptophan, 4-hydroxy-l-tryptophan, 5-hydroxy-l-tryptophan, l-tyrosine, l-phenylalanine, l-histidine and 3,4-dihydroxy-l-phenylalanine (l-DOPA). Reactions with heat-inactivated enzyme were used as negative controls. IasA accepted l-tryptophan and its hydroxy-derivatives (Fig. 4) while d-tryptophan was only minimally turned over and l-histidine was not accepted altogether. As l-tryptophan most likely represents the physiologically relevant substrate, its turnover was set to 100%. Highest turnover was found with 5-OH-l-tryptophan (132%) while l-DOPA, l-phenylalanine and l-tyrosine were turned over to a lesser extent (68, 61, and 43%, respectively). This substrate profile distinguishes IasA from PcDHPAAS, which was previously described as l-3,4-dihydroxyphenylacetaldehyde synthase [32]. Optimum turnover with IasA occurred at pH 9.0 in TRICIN buffer (Additional file 1: Figure S16) within a temperature plateau of 30–34 °C (Additional file 1: Figure S17). To verify indole-3-acetaldehyde as the IasA product, the reactions were treated with sodium borohydride which reduces the aldehyde to tryptophol. In the reactions, but not in the controls, a new chromatographic signal appeared at the same retention time as the synthetic tryptophol standard (t_R_ = 3.9 min, Fig. 5) with the matching mass to charge ratio (*m*/*z* 162.1 [M + H]^+^). Therefore, we unambiguously identified *P. mexicana* IasA as indole-3-acetaldehyde synthase, which represents the first characterized microbial acetaldehyde synthase accepting l-tryptophan as main substrate.

### Comparison of indoleamine-2,3-dioxygenases

The second gene whose transcription decreases as psilocybin is produced encodes an indoleamine-2,3-dioxy-genase (IDO). Typically, the Agaricomycotina encode three types of IDOs (a-c) that share a common phylogenetic origin. However, some of the genes can be absent or duplicated, depending on the species [33], and variation occurs even within the genus *Psilocybe*. Both *P. cubensis* and *P. mexicana* each encode one IdoA (type a) and IdoC (type c) enzyme. However, unlike *P. cubensis*, the sister species *P. mexicana* lacks genes for IdoB enzymes (type b, Additional file 1: Figure S18). *P. mexicana* IdoA and IdoC are equivalent to the counterparts in *P. cubensis* (Additional file 1: Table S5). In contrast, *P. cubensis* encodes two type b IDOs, whose genes were found upregulated in carpophores. Some fungal representatives, i.e., type c IDOs, show very low catalytic activity and their meta-bolic role is still unclear [33]. We suggest it is IdoA in *P. mexicana* that is primarily involved in l-tryptophan metabolism, as it is downregulated up to 350-fold under psilocybin production conditions (Additional file 1: Table S3, Figures S11 and S18). This transcriptional pattern correlates with the demand of l-tryptophan when psilocybin biosynthesis begins.

### Differential expression of tryptophan metabolism genes in *Psilocybe* spp

**Fig. 6:**
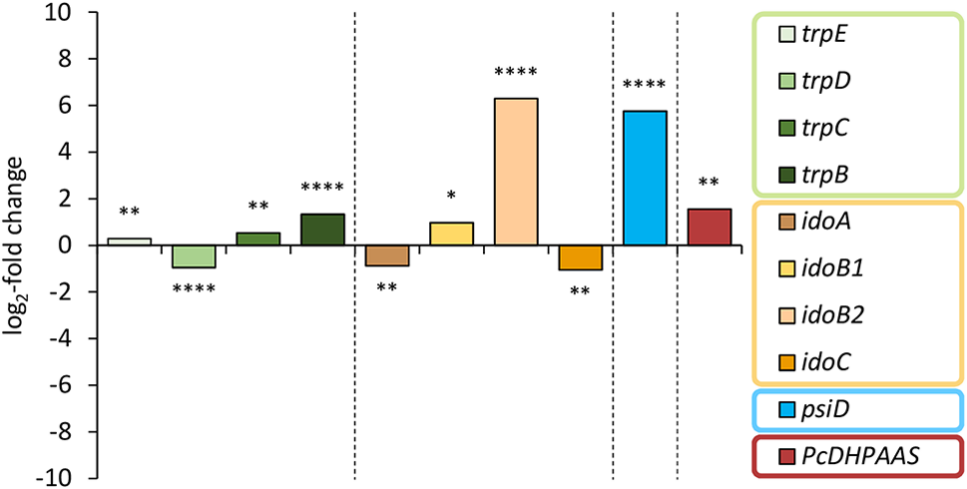
Expression analysis of selected genes involved in the tryptophan metabolism in *P. cubensis*. The RNA-Seq raw reads of mycelial and carpophore samples from Torrens-Spence et al. [32] were mapped and DESeq2-analyzed using Geneious Prime software. Genes that are upregulated in carpophores versus mycelium show positive log_2_-fold changes. Asterisks represent the calculated *p*-values: * 0.05 < *p*_adj_; ** 1∙10^− 10^ < *p*_adj_ ≤ 0.05; ** 1∙10^− 100^ < *p*_adj_ ≤ 1∙10^− 10^; **** *p*_adj_ ≤ 1∙10^− 100^. Color coding: green – tryptophan biosynthesis, orange/brown – tryptophan degradation, blue – psilocybin biosynthesis, maroon – aromatic acetaldehyde synthesis

The transcriptional dynamics of pertinent genes in *P*. *mexicana* carpophores was compared with prior data from *P. cubensis* mushrooms [32]. Surprisingly and contrasting *P. mexicana*, most of the investigated *P. cubensis* genes (Additional file 1: Table S6) related to l-tryptophan metabolism showed only marginal up or down regulation. The transcriptional changes of the genes coding for the tryptophan biosynthesis enzymes TrpE, TrpD, TrpC and TrpB, the indoleamine-2,3-dioxygenases IdoA, IdoB1 and IdoC and the aromatic acetaldehyde synthase PcDHPAAS range between − 2.1-fold and + 2.9-fold (log_2_-fold − 1.1 and + 1.6, Fig. 6, Additional file 1: Table S7). However, both species showed the pronounced regulation of *psiD* (54-fold and 170-fold for *P. cubensis* and *P. mexicana*, respectively, log_2_-fold values: 5.8 and 7.4). Another putative indoleamine-2,3-dioxygenase gene in *P. cubensis*, referred to as *idoB2* and for which a homolog does not exist in *P. mexicana*, was found to be 78-fold upregulated in *P. cubensis* carpophores (log_2_-fold 6.3), whereas either of the investigated *ido* genes of *P. mexicana* was downregulated. The expression pattern of the homologous genes encoding aromatic acetaldehyde synthases *(PcDHPAAS* in *P. cubensis*, log_2_-fold + 1.6; and *iasA* in *P. mexicana*, log_2_-fold − 3.0) is also diverging between the two investigated representatives of the *Psilocybe* genus. The phenomenon of oppositely regulated enzymes PcDHPAAS in *P. cubensis* and IasA in *P. mexicana* likely reflects the respective substrate preferences. Without downregulation, the latter enzyme would compete with PsiD for its substrate while the substrate of the former enzyme, l-DOPA, does not interfere. Hence, regulation of *PcDHPAAS* does not need to be adjusted relative to the l-tryptophan-requiring enzyme PsiD.

## Discussion

To ensure adequate supply of building block substrates and cofactors for enzymatic reactions, natural product pathways closely root in the cell’s central metabolism. The specialized purpose of the often bioactive and highly functionalized natural products, along with the demand for substrates of the central metabolism require that their assembly is a genetically tightly regulated process. Previous research predominantly emphasized ascomycetes and identified various levels of regulation. These include epigenetic modification as well as pathway-specific and global transcriptional control, e.g., by the prototypical pathway-specific regulator AflR for aflatoxin biosynthesis, the global regulator LaeA, or the regulatory circuits around penicillin biosynthesis [34–38]. Little is known about natural product pathway regulation in basidiomycetes, yet a correlation of blue light exposure and posttranscriptional regulation by light-dependent splicing has been shown [39].

Metabolic flux is a second important aspect of how central and secondary metabolism interface and contribute to regulation. Penicillin biosynthesis is arguably among the most prominent and best investigated examples. The analysis of central and amino acid metabolism in *Penicillium chrysogenum* revealed that the metabolic flux toward l-cysteine and l-valine strongly increases under penicillin production conditions to supply these amino acids as pathway substrates. Furthermore, an increased flux through the tricarboxylic acid cycle and the pentose phosphate pathway were observed to supply the energy-intensive synthetase reaction with ATP and the NADPH-intensive l-cysteine biosynthesis with reduction equivalents [40]. Likewise, production of the pharmaceutically invaluable polyketide lovastatin was enhanced in a genetically engineered *Aspergillus terreus* [41]. By overexpressing the gene for the acetyl-CoA carboxylase in *A. terreus*, an increased malonyl-CoA supply was offered to the lovastatin polyketide synthases, resulting in enhanced product titers.

This substantial body of research related to the metabolic flux for important ascomycete products is contrasted by our only rudimentary knowledge for basidiomycetes. For these, it has remained largely shrouded how natural product pathways are regulated and how the substrate supply is optimized to support a particular pathway. In the case of psilocybin, an interplay between primary metabolism and natural product biosynthesis has been reported for *P. cubensis* [8]. Adenosine kinase AdoK and *S*-adenosyl-l-homocysteine hydrolase (SahH) directly or indirectly remove the methyltransferase-inhibiting second product *S*-adenosyl-l-homocysteine and regenerate *S*-adenosyl-l-methionine (SAM), hence supporting the SAM-dependent methyltransfer as the final biosynthetic step. However, little is known about how the supply and degradation of the substrate l-tryptophan is genetically regulated except for the gene encoding the previously characterized tryptophan synthase TrpB [22], that is six-fold upregulated in carpophores of *P. cubensis*, compared to vegetative mycelium [8]. Furthermore, regulators that bind to promoters of genes encoding pathway and catabolic genes of l-tryptophan are unknown for the genus *Psilocybe*. In the medicinal mushroom *Ganoderma lucidum*, the basic leucine zipper (bZIP) transcription factor GCN4 serves as a master regulator for amino acid biosynthesis [42], which confirms earlier findings with *Saccharomyces cerevisiae* and Aspergilli, where *cpcA* encodes the gene homologous to *S. cerevisiae GCN4* and e.g., controls *trpB* expression [20, 43, 44]. *P. mexicana* encodes three genes homologous to *GCN4*. Only one of these (Additional file 1: Sequence data 1) showed an increase of transcription (log_2_-fold value 2.1) under psilocybin-producing conditions which might point to a function in upregulating amino acid metabolism. However, regulatory mechanisms other than on the transcriptional level appear possible as well. For example, import into nucleus [45, 46], posttranslational modification [47], or alternative splicing [39], although our *P. mexicana* transcriptomic data did not indicate the presence of differently spliced mRNA populations of the investigated genes. Hence, future work needs to establish the regulatory mechanism(s) of amino acid metabolic genes in *Psilocybe*.

In addition to analyzing anabolism and substrate supply, our study design also covered catabolism, which revealed the role of IasA, the indole-3-acetaldehyde synthase of *P. mexicana.* A similar enzyme, PcDHPAAS of *P. cubensis*, was previously characterized but found to prefer l-DOPA over l-tryptophan as substrate [32]. This finding underscores, once more, that subtle yet relevant differences between these closely related species and their enzymatic repertoire exist. Investigation of IasA is warranted for two reasons. First, it represents the first characterized microbial indole acetaldehyde synthase. Furthermore, it may play a role for chemical ecology as it catalyzes a key reaction toward indole acetic acid. This microbial, insect and auxin-type plant signal compound mediates interspecies interactions and insect gall formation [48, 49].

In conclusion, our results help understand the regulation of primary metabolism around tryptophan levels to optimize psilocybin-related secondary metabolic processes in *P. mexicana*. This knowledge will support efforts to control and increase the psilocybin content in mushrooms grown in certified facilities for legitimate purposes without any genetic manipulation. As mushrooms are notoriously difficult to modify genetically and given the status of psilocybin as a candidate drug to potentially treat major depressive disorders, the outcome of our study may promote biotechnology with *Psilocybe*. Beyond this particular metabolite and genus, our current work has pilot character as it addresses, for the first time, that mushrooms match primary and secondary metabolism through a coordinated regulation of anabolic and catabolic routes.

## Methods

### Materials and general procedures

Chemicals, media ingredients, and solvents were purchased from Carl Roth, Sigma-Aldrich, and VWR. Oligonucleotides were synthesized by Integrated DNA Technologies and are listed in Additional file 1: Tables S8 and S9. Restriction enzymes were purchased from NEB. Procedures to handle and modify DNA (extraction from agarose gels, restriction, dephosphorylation, ligation, and plasmid isolation) followed the manufacturers’ instructions (Macherey-Nagel, NEB).

### Microbial strains and growth conditions

*Psilocybe mexicana* SF013760 was maintained on malt extract peptone (MEP) agar plates (per liter: 30 g malt extract, 3 g peptone, 18 g agar, pH 5.5). To collect biomass from liquid cultures for nucleic acid extraction, *P. mexicana* was cultivated for 7 days in liquid MEP medium at 25 °C and 140 rpm. To find conditions suitable for RNA-Seq analysis, *P. mexicana* was precultured in 450 mL FB3G medium (per liter: 10 g malt extract, 10 g glucose, 5 g yeast extract, 3 g peptone, 0.1 g KH_2_PO_4,_ pH 5.5) for 7 days at 21 °C and 180 rpm. The preculture was dispersed and 10 mL each were used to inoculate 150 mL of different media. Selected media were: FB3G, MEP, BNM (as described in [19]), FB5B (similar to BNM but d-glucose increased to 7.5 g, and 6 g d-galactose per liter as additional carbon source), FB3B (similar to FB5B but yeast extract increased to 5 g per liter). The cultivation was continued for 7 days at 21 °C, 180 rpm in sextuplicates. Carpophore formation was induced as described [50]. Fungal biomass was collected, filtered through Miracloth (Merck) and washed with water if harvested from a liquid culture, shock-frozen in liquid nitrogen and lyophilized prior to nucleic acid or metabolite extraction. *Escherichia coli* KRX (Promega) was used for routine cloning, plasmid propagation and heterologous production of IasA. For cultivation of *E. coli*, LB medium (per liter: 5 g yeast extract, 10 g tryptone, 10 g NaCl, and 18 g agar if applicable) supplemented with 50 µg mL^− 1^ kanamycin sulfate was used. For heterologous production, 2 × YT medium (per liter: 10 g yeast extract, 20 g tryptone, 5 g NaCl) was used instead of LB medium.

### Nucleic acid isolation, first strand synthesis and qRT-PCR

Genomic DNA was isolated following a described protocol with a slight modification (isopropanol instead of ethanol precipitation) [51]. RNA isolation, reverse transcription, and qRT-PCR were performed as described [8, 52, 53]. The housekeeping reference gene *enoA*, encoding enolase, served as internal standard. Oligonucleotides with a primer efficiency of at least 90% were used for qRT-PCR (Additional file 1: Table S8). Gene expression levels were determined as described [54].

### RNA-Seq of *P. mexicana*

RNA was isolated from three biological replicates of *P. mexicana* grown in BNM and FB3G liquid medium as well as from carpophores produced in an axenic laboratory culture. RNA-Seq and parts of the bioinformatic analysis including the differential gene expression analysis, was performed by GENEWIZ. Sequences of 2 × 150 bp paired end reads were generated on an Illumina NovaSeq platform. Sequence fastq files were trimmed using Trimmomatic (v.0.36) [55] and mapped to the respective genome (GenBank: GCA_023853805.1) using the STAR aligner (v.2.5.2b) [56]. Unique gene hit counts were calculated using featureCounts [57] from the Subread package (v.1.5.2) [58]. Differential gene expression analysis was performed using DESeq2 [59]. log_2_-fold changes and *p*-values were generated by applying the Wald test [60]. The Benjamini Hochberg method [61] was used to calculate adjusted *p*-values. Trinity (v2.13.2) was used for RNA-Seq *de novo* assembly applying the standard settings [62, 63].

### Expression analysis of *P. cubensis* RNA-Seq raw reads with Geneious Prime software

The raw data published by Torrens-Spence et al. [32] (NCBI SRA: SRR7028478 and SRR7028479) was mapped to the *P. cubensis* genome (GenBank: GCA_017499595.2). The expression levels were calculated and compared with the Geneious method to measure the differential expression. As a result, log_2_-fold change values and *p* values were obtained (Fig. 6, Additional file 1: Table S7).

### Phylogenetic analyses of indoleamine-2,3-dioxygenases

Amino acid sequences were aligned using ClustalW2 [64] implemented in MEGA X software (v. 10.2.6) [65]. The evolutionary history was inferred by the Maximum Likelihood method and Le_Gascuel_2008 model [66]. A phylogenetic tree was constructed using the Maximum Likelihood method and the Jones-Taylor-Thornton model [67] and 1000 bootstrap replications [68].

### Protein structure prediction

Aromatic acetaldehyde synthase modeling was performed with AlphaFold2 [69] and was superimposed using ChimeraX [70, 71] and *Arabidopsis thaliana* phenylacetaldehyde synthase (PDBe 6eei [30]), as reference (Additional file 1: Figure S15).

### Heterologous production of IasA

The *iasA* coding sequence was PCR-amplified (Additional file 1: Table S10, PCR method A) from *P. mexicana* cDNA using oligonucleotides oPS628/629 (Additional file 1: Table S9). The agarose gel-purified fragment was ligated to the *Nco*I-*Xho*I-restricted and dephosphorylated (QuickCIP, NEB) plasmid pET28a using the NEBuilder HiFi DNA Assembly Cloning Kit (NEB) to yield expression plasmid pPS66. Correct assembly of insert and vector was verified by colony PCR (Additional file 1: Table S10, PCR method B), analytical restriction digests and DNA sequencing (GENEWIZ Inc.). IasA was produced in *E. coli* KRX × pPS66 essentially as described [27]. The protein was concentrated on an Amicon Ultra-15 centrifugal filter and eluted with 50 mm sodium phosphate buffer (pH 7.5). Protein concentrations were determined using the Pierce BCA-Protein Assay Kit (Thermo). The protein production was verified by SDS-polyacrylamide gel electrophoresis (SDS-PAGE) (Additional file 1: Figure S13).

### In vitro aldehyde formation assays

Aldehyde formation by IasA was monitored using a photometric assay and Brady’s reagent (2,4-dinitrophenylhydrazine, 2,4-DNPH) [31]. As described in [72], the freshly prepared detection solution consisted of 0.1% (w/v) 2,4-DNPH dissolved in MeOH with 1% (v/v) sulfuric acid. 100 µL of ice-cold detection solution were used to stop enzymatic reactions with the same volume following a 20 h incubation at 25 °C. Product formation was detected photometrically by measuring the absorption at λ = 500 nm (and 800 nm as reference wavelength) in a CLARIOstar plate reader (BMG LABTECH). Control reactions without substrates, without enzyme, neither with substrate nor with enzyme, or with heat-inactivated enzyme were run in parallel. The assay was performed twice in triplicates in 50 mm buffer (sodium phosphate, pH 7.5) with 1 mm of the respective substrate, 0.1 mm pyridoxal 5′-phosphate (PLP) and hexahistidine-tagged IasA at a final concentration of 13 µm.

### UHPLC-MS analysis of tryptophol formation in vitro

The assays were performed in triplicate at 25 °C for 20 h in 50 mm sodium phosphate buffer (pH 7.5) with 1 mm l-tryptophan, 0.1 mm pyridoxal 5′-phosphate (PLP) and hexahistidine-tagged IasA at a final concentration of 1 µm in a final volume of 50 µL. Reactions with heat-inactivated enzyme served as negative control. To analyze aldehydes reliably by high-performance liquid chromatography (HPLC), every reaction was stopped with 200 µL of sodium borohydride-saturated ethanol solution for reduction [29, 30, 73]. Formic acid (250 µL 0.8 m) was added after 5 min incubation at room temperature to decompose remaining borohydride and for an acidic pH (pH 4 to 5). Reactions were frozen in liquid nitrogen and subsequently lyophilized. The samples were dissolved in 200 µL methanol, centrifuged (10 min, 20,000 × g), and the supernatants were chromatographically analyzed by measuring areas under curves (AUCs) of extracted ion chromatogram (EIC) peaks. To determine optimal reaction conditions, the incubation time was shortened to 2 h and the final concentration of enzyme was increased to 2 µm. The pH was varied between 5 and 11 (5.0 to 6.5 in citrate, 6.0 to 8.0 in sodium phosphate, 7.5 to 9.0 in TRICIN, 8.5 to 10.0 in CHES, 9.5 to 11.0 in CAPS buffers) and the temperature was varied between 14 and 50 °C (TRICIN pH 9.0).

### Size exclusion chromatography

To verify that IasA is a homodimer, fast protein liquid chromatography (FPLC, Äkta Pure 25, GE Healthcare) equipped with a Superdex 200 increase 10/300 GL column with 24 mL bed volume was used. Binding and elution were performed at a flow of 0.5 mL min^− 1^ (i) with 50 mm sodium phosphate, 150 mm NaCl, pH 7.2 or (ii) with additional 6 M urea (denaturing conditions). Chromatograms were recorded at λ = 280, 340 and 400 nm.

### Chemical synthesis of tryptophol

The synthesis of tryptophol (2-(indol-3-yl)ethanol) was performed as described [74]. NMR spectroscopic data is listed in the supplementary material, ^1^H and ^13^C NMR spectra are shown in Additional file 1: Figures S19 and S20.

### Liquid chromatography and mass spectrometry

Methanol extracts of in vitro experiments with IasA were subjected to UHPLC-MS analysis on an Agilent 1290 Infinity II instrument, interfaced to an Agilent 6130 single quadrupole mass detector, operated in alternating positive/negative mode. The chromatograph was fitted with an Ascentis Express F5 column (100 × 2.1 mm, 2.7 μm particle size). Separation was at 35 °C. Solvent A was 0.1% formic acid in water, solvent B was methanol. A linear gradient at a flow rate of 0.4 mL min^− 1^ was applied: within 8 min from 10 to 100% B, held for 2 min at 100%. Diode array detection was performed between λ = 200–600 nm. Chromatograms were extracted at λ = 205, 224, 254, 269 and 280 nm. To analyze methanolic extracts of *P. mexicana* mycelium, the same instrument, equipped with a Luna Omega Polar C18 column (50 × 2.1 mm, 1.6 μm particle size) was used. Solvent A was 0.1% formic acid in water, solvent B was acetonitrile. The flow was 1 mL min^− 1^. The gradient was: initially 1% B, increase to 5% B within 3 min, to 100% B within further 1 min, held at 100% B for 2 min. Chromatograms were extracted at λ = 254 and 280 nm.

### Electronic supplementary material

Below is the link to the electronic supplementary material.

Supplementary Material 1

## Data Availability

The genomic sequence of *Psilocybe mexicana* has been published [75] and is accessible under GenBank ID GCA_023853805.1. The raw RNA-Seq reads have been deposited in NCBI SRA (PRJNA1093255). The cDNA sequence of *iasA* is deposited under the GenBank accession number PP316613.
