# Supplementary material for: Genetic regulation of l-tryptophan metabolism in Psilocybe mexicana supports psilocybin biosynthesis
Source: Fungal Biol Biotechnol. 2024 Apr 25;11:4. doi: 10.1186/s40694-024-00173-6 (PMC11046786; doi:10.1186/s40694-024-00173-6)
Supplement: Supplementary file 1 — Supplementary Material 1 [file 40694_2024_173_MOESM1_ESM.pdf]

Genetic Regulation of L-Tryptophan Metabolism in *Psilocybe mexicana* Supports Psilocybin Biosynthesis

Supporting information

Table of Contents

Experimental procedures. Chemical synthesis of tryptophol (2-(indol-3-yl)ethanol) 2
Table S1. Comparison of gene expression in *P. mexicana*. 3
Table S2. Selected *P. mexicana* genes for differential expression analysis 4
Table S3. Differential expression of selected genes involved in the tryptophan metabolism in *P. mexicana* 5
Table S4 Sequence identities and similarities of AASs and AAADs 6
Table S5 Sequence identities and similarities of indoleamine-2,3-dioxygenases 7
Table S6 Selected *P. cubensis* genes for differential expression analysis 8
Table S7. Differential expression of selected genes involved in the tryptophan metabolism in *P. cubensis* 9
Table S8. Oligonucleotides used for expression analysis by qRT-PCR 10
Table S9. Oligonucleotides used for cloning, colony PCRs, and DNA sequencing 11
Table S10. PCR methods 12
Figure S1. L-Tryptophan biosynthesis genes in selected organisms. 13
Figure S2. Relative psilocybin content in *P. mexicana* mycelium grown submers in different media 14
Figure S3. Chromatographic analysis of *P. mexicana* mycelium extracts 15
Figure S4. RNA-Seq analysis with *P. mexicana* transcripts (I) 16
Figure S5. RNA-Seq analysis with *P. mexicana* transcripts (II) 17
Figure S6. RNA-Seq analysis with *P. mexicana* transcripts (III) 18
Figure S7. Venn diagram of hit counts 19
Figure S8. Bi-clustering of top 30 p_{adj}-value significant differentially expressed genes (I) 20
Figure S9. Bi-clustering of top 30 p_{adj}-value significant differentially expressed genes (II) 21
Figure S10. Bi-clustering of top 30 p_{adj}-value significant differentially expressed genes (III) 22
Figure S11. Differential expression of selected genes involved in the tryptophan metabolism in *P. mexicana* ... 23
Figure S12. Sequence alignment of selected aromatic acetaldehyde synthases (IasA) and aromatic amino acid decarboxylases (AAAD) 24
Figure S13. SDS-polyacrylamide gel electrophoresis of C-terminally hexahistidine-tagged *P. mexiana* IasA 25
Figure S14. Size exclusion chromatography of native and denatured IasA 26
Figure S15. Structure prediction of *P. mexicana* IasA 27
Figure S16. pH optimum of *P. mexicana* IasA 28
Figure S17. Temperature optimum of *P. mexicana* IasA 28
Figure S18. Analysis of indoleamine-2,3-dioxygenase genes and enzymes 29
Figure S19. ^{1}H NMR spectrum of synthesized 2-(indol-3-yl)ethanol (tryptophol) 30
Figure S20. ^{13}C NMR spectrum of synthesized 2-(indol-3-yl)ethanol (tryptophol) 31
Sequence data 1. *P. mexicana* gene homologous to *Ganoderma lucidum gcn4* 32

References. 33

Experimental procedures

Chemical synthesis of tryptophol (2-(indol-3-yl)ethanol). The synthesis of 2-(indol-3-yl)ethanol, from Indole-3-acetic acid was performed as described [1]. The NMR spectra (Figure S19 and S20) are in accordance with the cited literature [1].

^{1}H NMR (300 MHz, $CDCl_3$) δ 1.58 (br s, 1H, O*H*), 3.05 (t, *J* = 6.3 Hz, 2H, CH_2CH_2OH), 3.92 (dt, *J* = 2.0, 6.3 Hz, 2H,CH_2OH), 7.10 (s, 1H, Ar*H*), 7.14 (ddd, *J* = 7.8, 7.5, 1,1 Hz, 1H, Ar*H*), 7.22 (ddd, *J* = 8.1,7.6, 1.1 Hz, 1H, Ar*H*), 7.39 (d, *J* = 8.1 Hz, 1H, Ar*H*), 7.63 (d, *J* = 7.7 Hz, 1H, Ar*H*), 8.05(br s, 1H, N*H*) ppm; ^{13}C NMR (300 MHz, $CDCl_3$) δ 28.9, 62.8, 111.3, 112.4, 119.0, 119.6, 122.4, 122.6, 127.5, 136.6 ppm

Table S1. Comparison of gene expression in *P. mexicana*. The log_2 ratio, calculated with the DESeq2 method, shows the fold change in expression between two sample conditions. If the log_2-fold change is positive, genes are upregulated in the second listed sample condition. FK = carpophores; p_{adj} = Benjamini-Hochberg adjusted *p*-value.

**Compared conditions**	**BNM vs. FB3G**	**FK vs. BNM**	**FK vs. FB3G**
total number of genes with calculated log_2-fold change	15950	16119	16287
upregulated (log_2-fold change > 1)	3114	4199	3949
nonregulated ($-1 \leq log_2$-fold change ≤ 1)	9375	8479	8488
downregulated (log_2-fold change < -1)	3461	3441	3850
significant ($p_{adj} < 0.05$)	9056	11780	10312
significantly upregulated (log_2-fold change > 1 and $p_{adj} < 0.05$)	2986	4171	3846
significantly downregulated (log_2-fold change < -1 and $p_{adj} < 0.05$)	3291	3382	3676

Table S2. Selected *P. mexicana* genes for differential expression analysis.

Gene	(Putatively) encoding	Gene ID	Contig	Reference for Megablast sequence search with Geneious software	E value
trpE	anthranilate synthase	g9613<br>g13752	tig00000211<br>tig00000235	*Coprinopsis cinerea okayama* anthranilate synthase XP_001833757.2<br>*Paxillus involutus* anthranilate synthase KIJ16135.1	0<br>0
trpD	anthranilate phosphoribosyltransferase	g3736	tig00000106	*Psilocybe cubensis* anthranilate phosphoribosyltransferase XP_047748016.1	2.59E-130
trpC	phosphoribosyl anthranilate isomerase + indole-3-gylcerol-phosphate synthase	g20402	tig00000291	*Dichomitus squalens N*-anthranilate isomerase TBU59718.1<br>*Dichomitus squalens* indole-3-glycerol phosphate synthase TBU32557.1	0<br>1.79E-77
trpB	tryptophan synthase	g338	tig00000015	*Psilocybe cubensis* tryptophan synthase AWK77754.1	0
ido	indoleamine-2,3-dioxygenase	g11833<br>g9709	tig00000219<br>tig00000212	*Dichomitus squalens* indoleamine-2,3-dioxygenase TBU55451.1<br>*Dichomitus squalens* indoleamine-2,3-dioxygenase TBU65517.1	2.69E-139<br>1.68E-94
psiD	tryptophan decarboxylase	g12620	tig00000230	*Psilocybe cubensis* tryptophan decarboxylase PsiD ASU62239.1	4.55E-14
iasA	aromatic acetaldehyde synthase	g20428	tig00000291	*Psilocybe cubensis* PcDHPAAS AYU58583	3.26E-142

Table S3. Differential expression of selected genes involved in the tryptophan metabolism in *P. mexicana*.

Gene	Gene ID	FB3G vs. FK		BNM vs. FK		FB3G vs. BNM	
		log_2-fold change	adjusted *p*-value	log_2-fold change	adjusted *p*-value	log_2-fold change	adjusted *p*-value
trpE1	g9613	1.45	2.84E-06	2.00	1.34E-56	-0.62	6.63E-02
trpE2	g13752	-0.78	3.52E-05	0.61	2.65E-03	-1.46	1.31E-11
trpD	g3736	3.39	9.74E-143	3.08	1.78E-174	0.24	1.54E-01
trpC	g20402	0.96	4.51E-03	2.17	1.65E-59	-1.28	1.61E-04
trpB	g338	3.14	1.21E-60	3.74	1.23E-250	-0.67	1.70E-03
idoA	g11833	-8.45	2.41E-29	-6.37	9.30E-19	-2.14	1.69E-10
idoC	g9709	-0.76	1.33E-01	-3.14	9.03E-61	2.32	6.23E-08
psiD	g12620	7.41	9.64E-236	4.49	9.42E-306	2.85	1.93E-33
iasA	g20428	-3.00	3.05E-70	-1.31	5.63E-13	-1.76	1.03E-21

Table S4. Sequence identities and similarities of AASs and AAADs. Pairwise identity [%]/pairwise positive [%]. AAS = aromatic acetaldehyde synthase, AAAD = aromatic amino acid decarboxylase, IasA = *Psilocybe mexicana* AAS (PP316613), PcDHPAAS = *Psilocybe cubensis* AAS (AYU58583), *At*AAS = *Arabidopsis thaliana* AAS (NP_849999), *Oe*AAS = *Olea europea* AAS (QJA07379), *Pc*AAS = *Petroselinum crispum* AAS (Q06086), *Rhc*AAS = *Rosa hybrid cultivar* AAS (ABB04522), *At*AAAD = *Arabidopsis thaliana* AAAD (AtTYD, NP_001078461), *Cr*AAAD = *Catharanthus roseus* AAAD (CrTDC, P17770), *Ps*AAAD = *Papaver somniferum* AAAD (TyDC9, AAC61842), *Os*AAAD = *Oryza sativa* AAAD (TDC, AK069031), *Tf*AAAD = *Thalictrum flavum* AAAD (TYDC1, AAG60665).

	**IasA**	***PcDHPAAS***	***At*AAS**	***Oe*AAS**	***Pc*AAS**	***Rhc*AAS**	***At*AAAD**	***Cr*AAAD**	***Ps*AAAD**	***Os*AAAD**	***Tf*AAAD**
**IasA**	100/100	80/85	40/56	38/54	38/53	38/54	39/53	36/53	35/53	35/52	36/54
PcDHPAAS		100/100	41/56	39/55	37/52	36/54	39/53	36/53	35/53	34/51	35/53
***At*AAS**			100/100	75/81	56/69	55/68	68/76	51/67	54/71	47/65	55/71
***Oe*AAS**				100/100	61/70	56/70	67/77	52/69	57/72	47/65	57/72
***Pc*AAS**					100/100	65/74	52/66	54/68	68/78	48/63	68/77
***Rhc*AAS**						100/100	52/67	55/69	67/77	46/63	67/78
***At*AAAD**							100/100	48/65	51/69	43/60	52/68
***Cr*AAAD**								100/100	55/70	54/70	55/71
***Ps*AAAD**									100/100	47/65	78/84
***Os*AAAD**										100/100	45/62
***Tf*AAAD**											100/100

Table S5. Sequence identities and similarities of indoleamine-2,3-dioxygenases. Pairwise identity [%]/pairwise positive [%]. The amino acid sequences of *P. mexicana* IdoA and IdoC are most similar to those of *P. cubensis* IdoA and IdoC, respectively (highlighted with brown and orange shade, respectively).

	***P. cubensis* IdoA**	***P. cubensis* IdoB1**	***P. cubensis* IdoB2**	***P. cubensis* IdoC**	***P. mexicana* IdoA**	***P. mexicana* IdoC**
***P. cubensis* IdoA**	100/100	47/57	41/52	28/44	72/75	29/43
***P. cubensis* IdoB1**		100/100	48/56	32/44	45/57	32/46
***P. cubensis* IdoB2**			100/100	28/43	39/52	27/42
***P. cubensis* IdoC**				100/100	30/45	62/68
***P. mexicana* IdoA**					100/100	30/43
***P. mexicana* IdoC**						100/100

Table S6. Selected *P. cubensis* genes for differential expression analysis. Genome Genbank accession: GCA_017499595.2.

Gene	(Putatively) encoding	Gene ID	Contig	Reference for Megablast sequence search with Geneious software	E value
trpE	anthranilate synthase	KAH9484046.1	CM039000.1	*Paxillus involutus* anthranilate synthase KIJ16135.1	0
trpD	anthranilate phosphoribosyltransferase	KAH9480391.1	CM039003.1	*Psilocybe cubensis* anthranilate phosphoribosyltransferase XP_047748016.1	0
trpC	phosphoribosyl anthranilate isomerase + indole-3-gylcerol phosphate synthase	KAH9485158.1	CM038999.1	*Dichomitus squalens* *N*-anthranilate isomerase TBU59718.1<br>*Dichomitus squalens* indole-3-glycerol phosphate synthase TBU32557.1	0<br>6.75E-100
trpB	tryptophan synthase	KAH9483885.1	CM039000.1	*Psilocybe cubensis* tryptophan synthase AWK77754.1	0
ido	indoleamine-2,3-dioxygenase	KAH9481074.1	CM039002.1	*Pleurotus ostreatus* hypothetical protein (indoleamine-2,3-dioxygenase) KDQ24310.1	1.47E-135
		KAH9481098.1	CM039002.1	*Dichomitus squalens* indoleamine-2,3-dioxygenase TBU55451.1	7.74E-134
		KAH9482090.1	CM039000.1	*Dichomitus squalens* indoleamine-2,3-dioxygenase TBU55451.1	6.77E-103
		KAH9483962.1	CM039000.1	*Dichomitus squalens* indoleamine-2,3-dioxygenase TBU65517.1	8.82E-119
psiD	tryptophan decarboxylase	KAH9476873.1	CM039007.1	*Psilocybe cubensis* tryptophan decarboxylase PsiD ASU62239.1	0
PcDHPAAS	aromatic acetaldehyde synthase	KAH9485139.1	CM038999.1	*Psilocybe cubensis* PcDHPAAS AYU58583	0

Table S7. Differential expression of selected genes involved in the tryptophan metabolism in *P. cubensis*. The RNA-Seq raw reads of mycelial and carpophore samples from Torrens-Spence et al. [2] were mapped and DESeq2-analyzed using Geneious Prime software. Genome Genbank accession: GCA_017499595.2.

**Gene**	**Gene ID**	**mycelium vs. carpophore**	
		**$\log_2$-fold change**	***p*-value**
trpE1	KAH9484046.1	0.29	1.90E-02
trpD	KAH9480391.1	-0.95	0.00E+00
trpC	KAH9485158.1	0.53	2.30E-04
trpB	KAH9483885.1	1.33	0.00E+00
idoA	KAH9481074.1	-0.88	4.20E-02
idoB1	KAH9481098.1	0.97	3.10E-01
idoB2	KAH9482090.1	6.29	0.00E+00
idoC	KAH9483962.1	-1.05	2.20E-04
psiD	KAH9476873.1	5.75	0.00E+00
PcDHPAAS	KAH9485139.1	1.55	1.70E-02

Table S8. Oligonucleotides used for expression analysis by qRT-PCR.

Oligonucleotide	5´-3´sequence	Target gene	Fragment size (gDNA) [bp]	Fragment size (cDNA) [bp]	Primer efficiency
oPS326	CGAAAGTAACCCTGCAGTTCAC	*psiD*	180	118	1.00
oPS267	CGGCGCTCTACGAAAGATAT				
oPS335	CGCAAGCGAAACGTATCAGA	*enoA*	174	118	0.94
oPS336	AGCCTCTTCAGGAGTTGAGA				
oPS608	ATACAACCACGCTATCGCTG	*trpE1*	175	119	0.94
oPS609	CGACGAGCATGATGTGTTCT				
oPS610	CATGGCTTGGAAAGTTCATGC	*trpE2*	215	215	0.97
oPS611	ACTGGACGTATGTTTTCCCG				
oPS612	TCAAGAGCATGGAGGAAACG	*idoA*	158	106	0.91
oPS613	TCCATGCTAAGACGAGATGAGC				
oPS614	GCGCTGGAGACAAAACATTG	*idoC*	167	108	0.96
oPS615	TTGACAAGCCAAGCAAGGAC				
oPS616	GTGGACAACATCCCTCAGATC	*iasA*	197	145	0.94
oPS617	GGACAAAGTCGTTTATGGCG				
oPS657	GCAGAATACGTCGTTGCAAC	*trpB*	171	119	1.02
oPS658	ATCTTCACCGCTTCCCAGAT				
oPS733	TCACTCGGATCCAAGGTGATA	*trpC*	189	189	1.03
oPS734	AATTAACACGGCGCCTACAC				
oPS745	GCTGCACATCCATCGGGT	*trpD*	169	169	1.01
oPS746	CGTCGTGCTGACATTGAACA				

Table S9. Oligonucleotides used for cloning, colony PCRs, and DNA sequencing.

Oligonucleotide	5´-3´sequence	Target	Purpose
T7-mod	CCCGCGAAATTAATACGACTCAC	pET28a	colony PCR and sequencing
T7-term	CTAGTTATTGCTCAGCGGT	pET28a	sequencing
oPS617	GGACAAAGTCGTTTATGGCG	*iasA*	colony PCR and sequencing
oPS628	AACTTTAAGAAGGAGATATACCATGGATATCGAACAATTTAGAAAAGCTG	*iasA*	cloning and expression of *iasA*
oPS629	AGTGGTGGTGGTGGTGGTGCTCGAGACCAACTATTGCCATTCCGTTC	*iasA*	cloning and expression of *iasA*

Table S10. PCR methods.

Method	Reaction Mix		Thermal cycling		
	Component	Volume [µL]	Temperature [°C]	Time	Cycles
A	2 × Xtreme Buffer	25	94	2 min	1
	2 mM dNTP Mix	10	98	10 s	36
	Primer forward 10 pmol μL^{-1}	1.5	60	30 s	
	Primer reverse 10 pmol μL^{-1}	1.5	68	1 min kb^{-1}	
	DNA template*	1	68	5-10 min	1
	KOD Xtreme Hot Start DNA Polymerase (1 U μL^{-1})	1	10	∞	
	dH_2O	to 50			
B	10 × DreamTaq Buffer	2	95	2 min	1
	10 mM dNTP Mix	0.4	95	30 s	31
	Primer forward 10 pmol μL^{-1}	0.4	60	30 s	
	Primer reverse 10 pmol μL^{-1}	0.4	72	1 min kb^{-1}	
	DNA template**	10	72	5-10 min	1
	DreamTaq Polymerase (5 U μL^{-1})	0.1	10	∞	
	dH_2O	to 20			

***DNA template: 1 µL of reverse transcription reaction (1:5 diluted with dH_2O), **10 µL water with resuspended *E. coli* cells (for colony PCR) or Gibson assembly reaction (1:10 diluted with dH_2O).

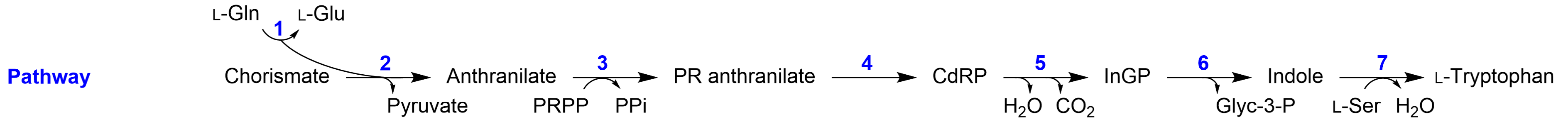

	G	E	D	F		C	B		A	
**Enzyme**		**Anthranilate synthase** EC:4.1.3.27	**Anthranilate PR transferase** EC:2.4.2.18	**PR anthranilate isomerase** EC:5.3.1.24		**InGP synthase** EC:4.1.1.48		**Tryptophan synthase** EC:4.2.1.20		
**Activity domain**	**G**	**E**	**D**	**F**		**C**	**B**		**A**	
**Organism**	Genes encoding enzymes									Reference
**Basidiomycetes**										
Psilocybe mexicana	*trpC*	*trpE*	*trpD*		*trpC*			*trpB*		this work and [4]
Psilocybe cubensis	*trpC*	*trpE*	*trpD*		*trpC*			*trpB*		this work and [2]
Coprinus cinereus	*trp2*	*trp3*	*trp4*		*trp2*			*trp1*		[5]
Cryptococcus neoformans	*trp3*	*trp2*	*trp4*		*trp3*			*trp5*		[6]
**Ascomycetes**										
Aspergillus fumigatus	*trpC*	*trpE*	*trpD*		*trpC*			*trpB*		[7]
Fonsecaea pedrosoi	*trpC*	*trpE*	*trpD*		*trpC*			*trpB*		[8]
Neurospora crassra	*trp1*	*trp2*	*trp4*		*trp1*			*trp3*		[9]
Saccharomyces cerevisiae	*trp3*	*trp2*	*trp4*	*trp1*		*trp3*		*trp5*		[10]
**Bacteria**										
Escherichia coli	*trpD*	*trpE*	*trpD*		*trpC*		*trpB*		*trpA*	[11,12]
Pseudomonas putida	*trpG*	*trpE*	*trpD*	*trpF*		*trpC*	*trpB*		*trpA*	[12,13]
Serratia marcescens	*trpG*	*trpE*	*trpD*		*trpC*		*trpB*		*trpA*	[12,14]
Clostridium acetobutylicum	*trpG*	*trpE*	*trpD*	*trpF*		*trpC*	*trpB*		*trpA*	[15]

Figure S1. L-Tryptophan biosynthesis genes in selected organisms. In *Psilocybe mexicana* and *P. cubensis, trpE* encodes anthranilate synthase and *trpD* anthranilate phosphoribosyl transferase. The gene *trpC* encodes a trifunctional enzyme: glutamine amidotransferase (G domain), phosphoribosyl anthranilate isomerase (F domain), and indole-3-glycerol phosphate synthase (C domain). Tryptophan synthase is encoded by *trpB* and is a homodimer, consisting of monomers composed of an α- and a β-domain, [3]. PR: phosphoribosyl; CdRP: 1-(*o*-Carboxyphenylamino)-1-deoxyribulose-5-phosphate; InGP: indole-3-glycerol phosphate; L-Gln: L-glutamine; L-Glu: L-glutamate; PRPP: phosphoribosyl diphosphate; PPi: inorganic pyrophosphate; Glyc-3-P: glyceraldehyde 3-phosphate; L-Ser: L-serine.

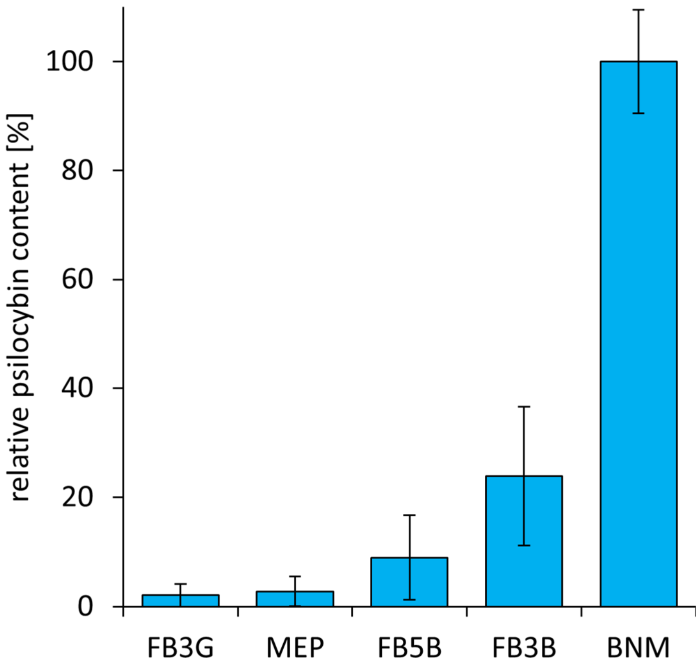

Figure S2. Relative psilocybin content in *P. mexicana* mycelium grown in different media as submers cultures. The psilocybin content was normalized to the dry weight and referenced to the highest value. Mean values and standard deviations (n=6) are shown.

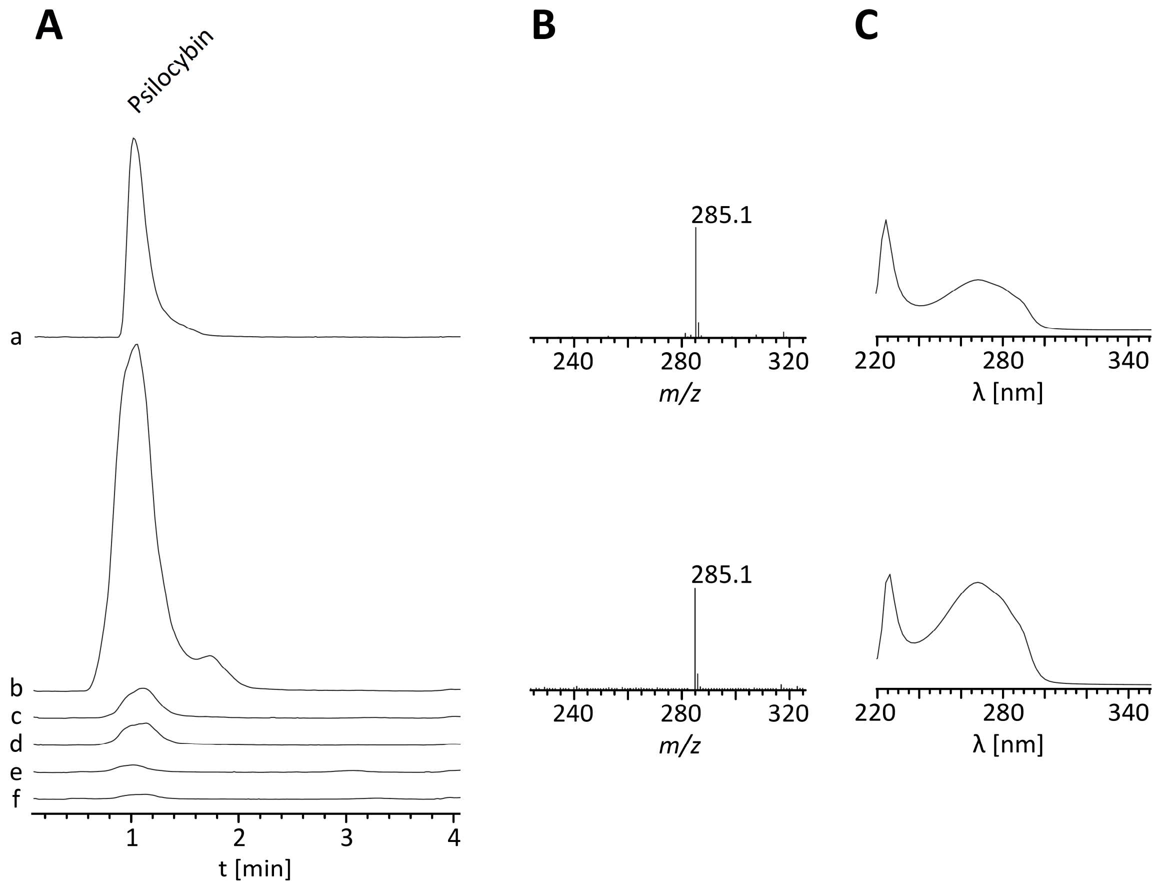

Figure S3. Chromatographic analysis of *P. mexicana* mycelium extracts. Shown are (**A**) extracted ion chromatograms (EICs) for *m/z* 285 $[M+H]^+$, and for traces a and b corresponding mass spectra (**B**), recorded in positive mode and (**C**) UV/Vis spectra of chromatographic signals of psilocybin. Trace a: psilocybin reference, traces b-f: extracts of *P. mexicana* mycelium grown in (b) BNM, (c) FB3B, (d) FB5B, (e) MEP and (f) FB3G medium.

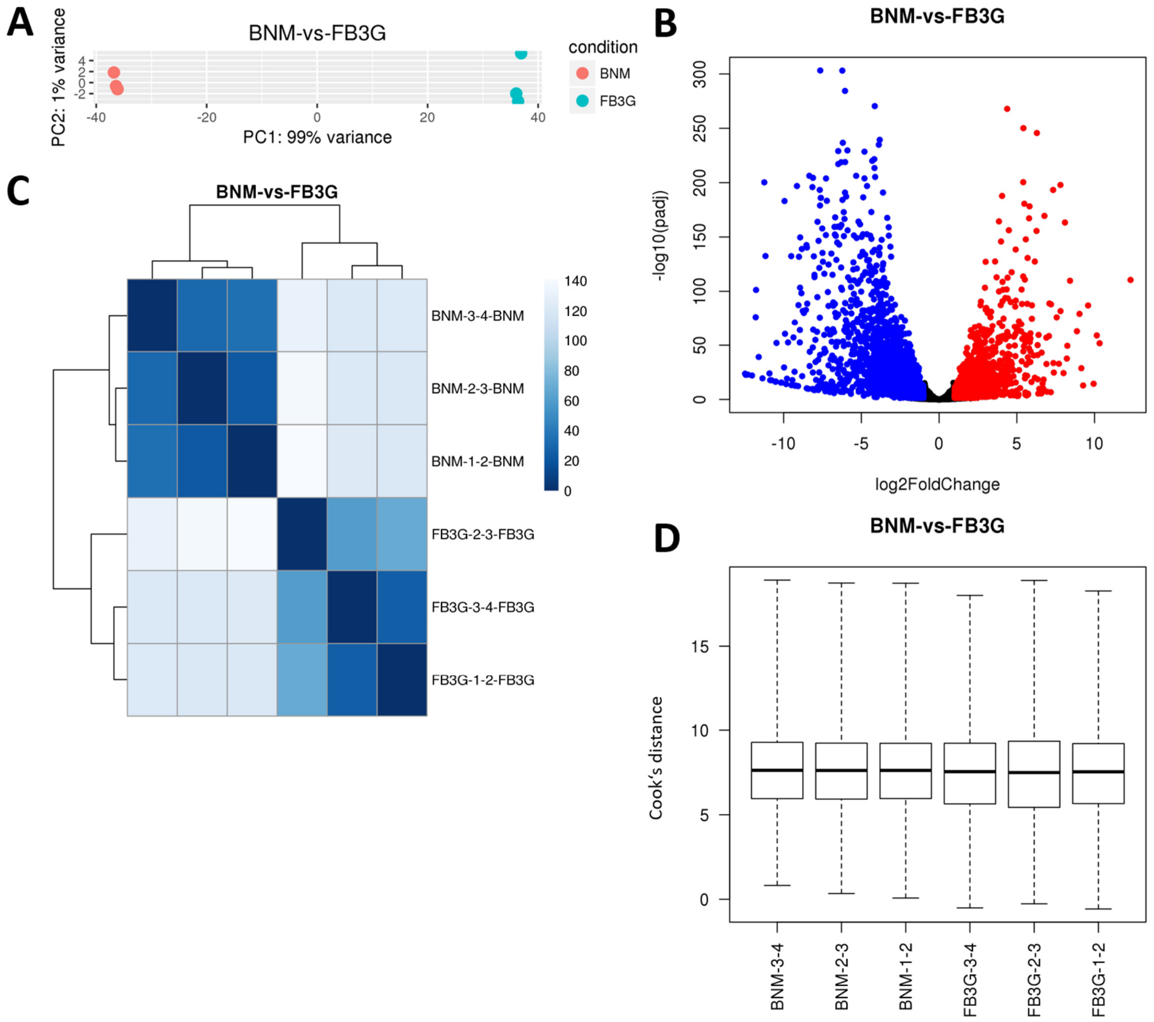

Figure S4. RNA-Seq analysis with *P. mexicana* transcripts (I). DESeq2 analysis was conducted with mycelium grown in BNM versus FB3G medium. (**A**) PCA plot. (**B**) Volcano plot. (**C**) Sample distance. (**D**) Boxplot of the Cook's distance of normalized data.

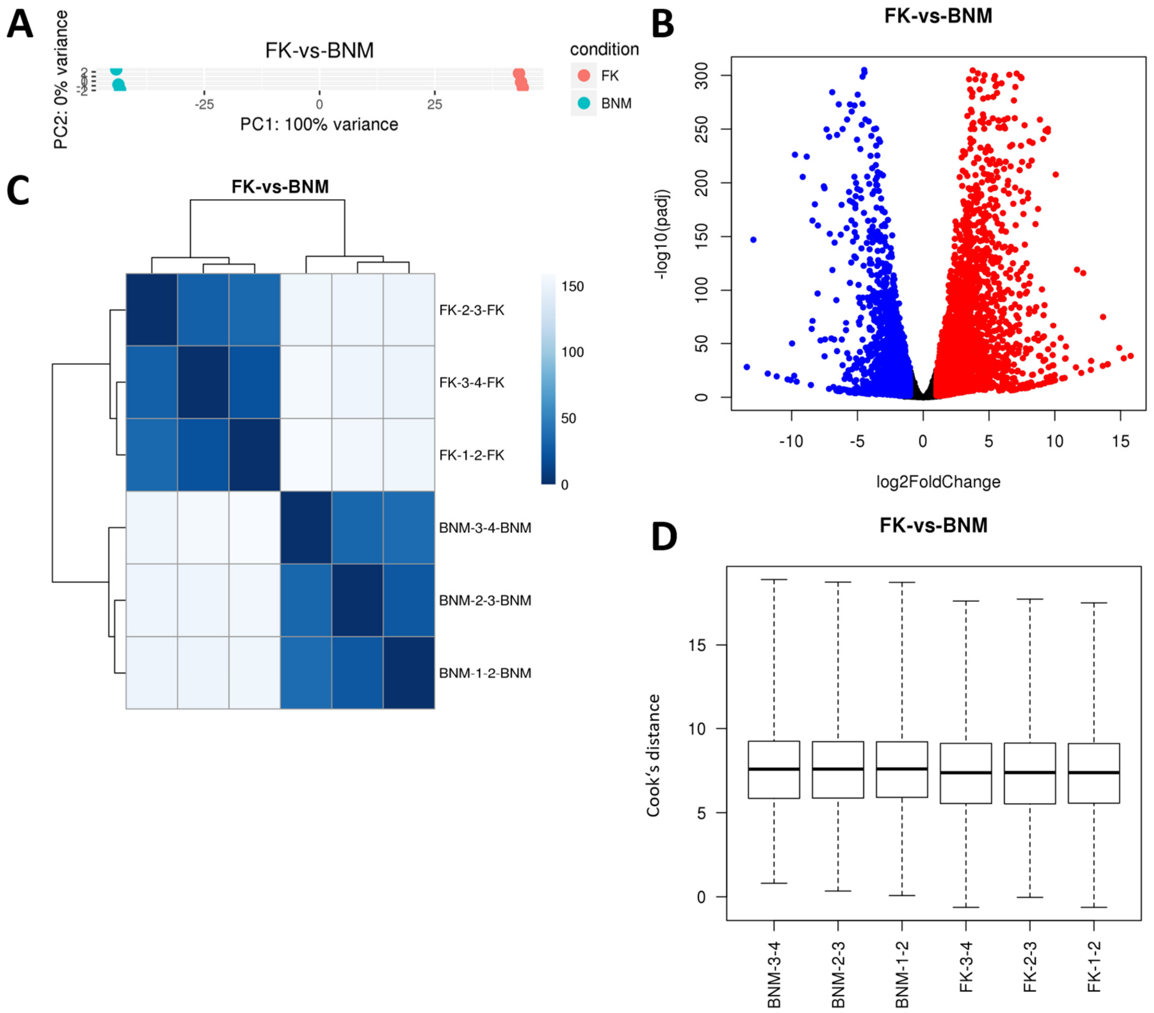

Figure S5. RNA-Seq analysis with *P. mexicana* transcripts (II). DESeq2 analysis was conducted with carpophores (FK) and vegetative mycelium, grown in BNM medium. (**A**) PCA plot. (**B**) Volcano plot. (**C**) Sample distance. (**D**) Boxplot of the Cook's distance of normalized data.

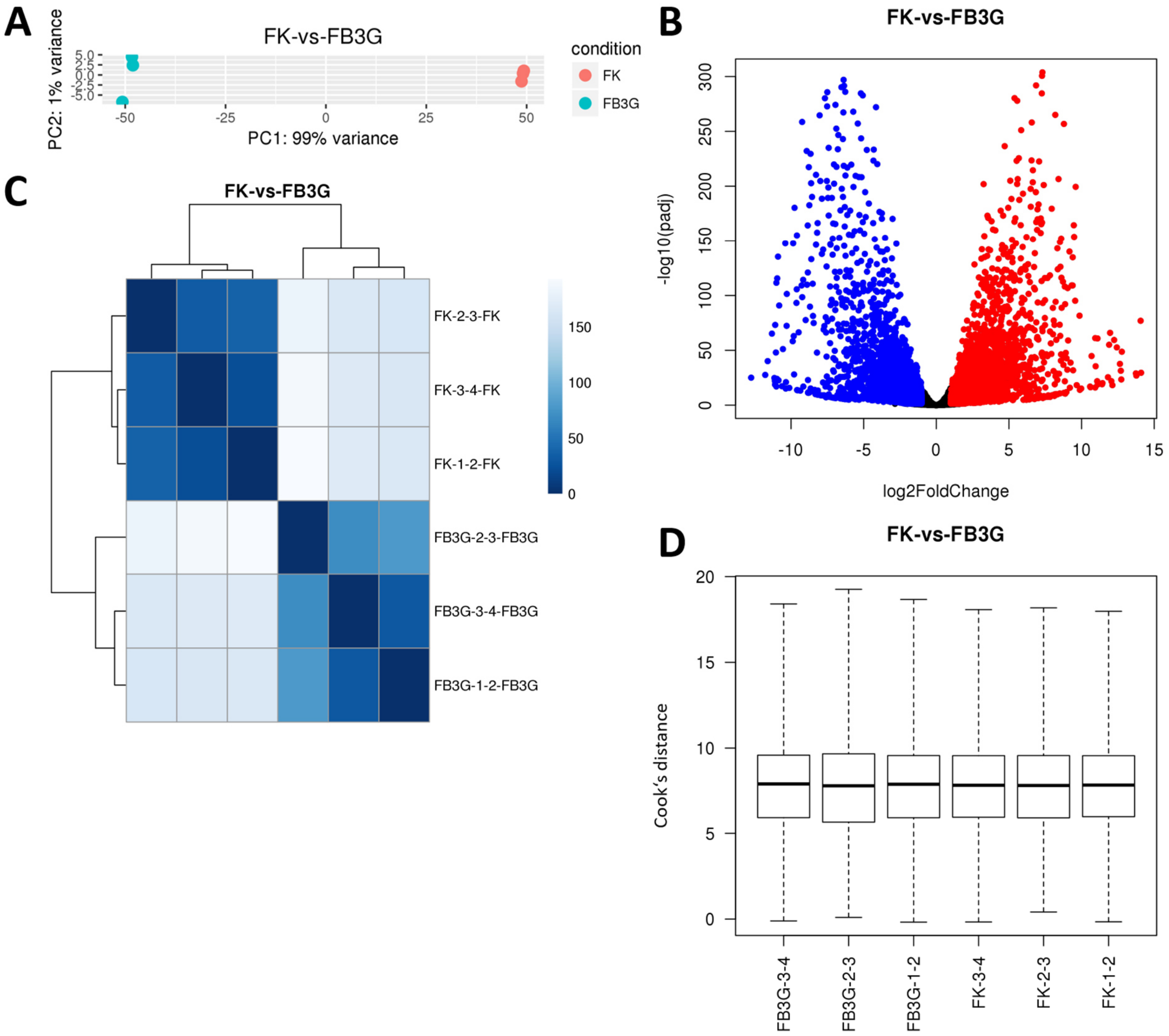

Figure S6. RNA-Seq analysis with *P. mexicana* transcripts (III). DESeq2 analysis was conducted with carpophores (FK) and vegetative mycelium, grown in FB3G medium. (**A**) PCA plot. (**B**) Volcano plot. (**C**) Sample distance. (**D**) Boxplot of the Cook's distance of normalized data.

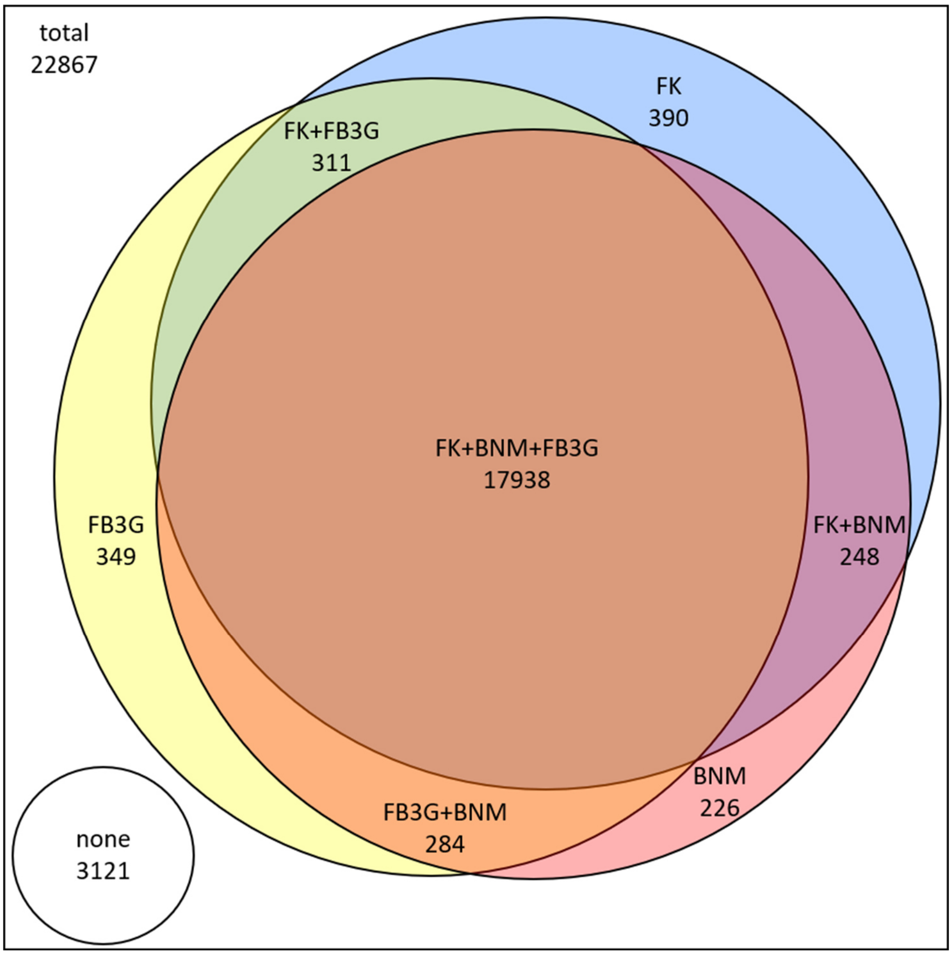

Figure S7. Venn diagram of hit counts. The growth condition-dependent expression of 22,867 annotated *P. mexicana* genes was analyzed. The conditions were mycelium grown in FB3G medium, mycelium grown in BNM medium, and carpophores (FK); each n=3. The diagram was generated based on the hit counts. If at least one of three biological replicates had a TPM (transcripts per million) value above 0, this was counted as gene expression.

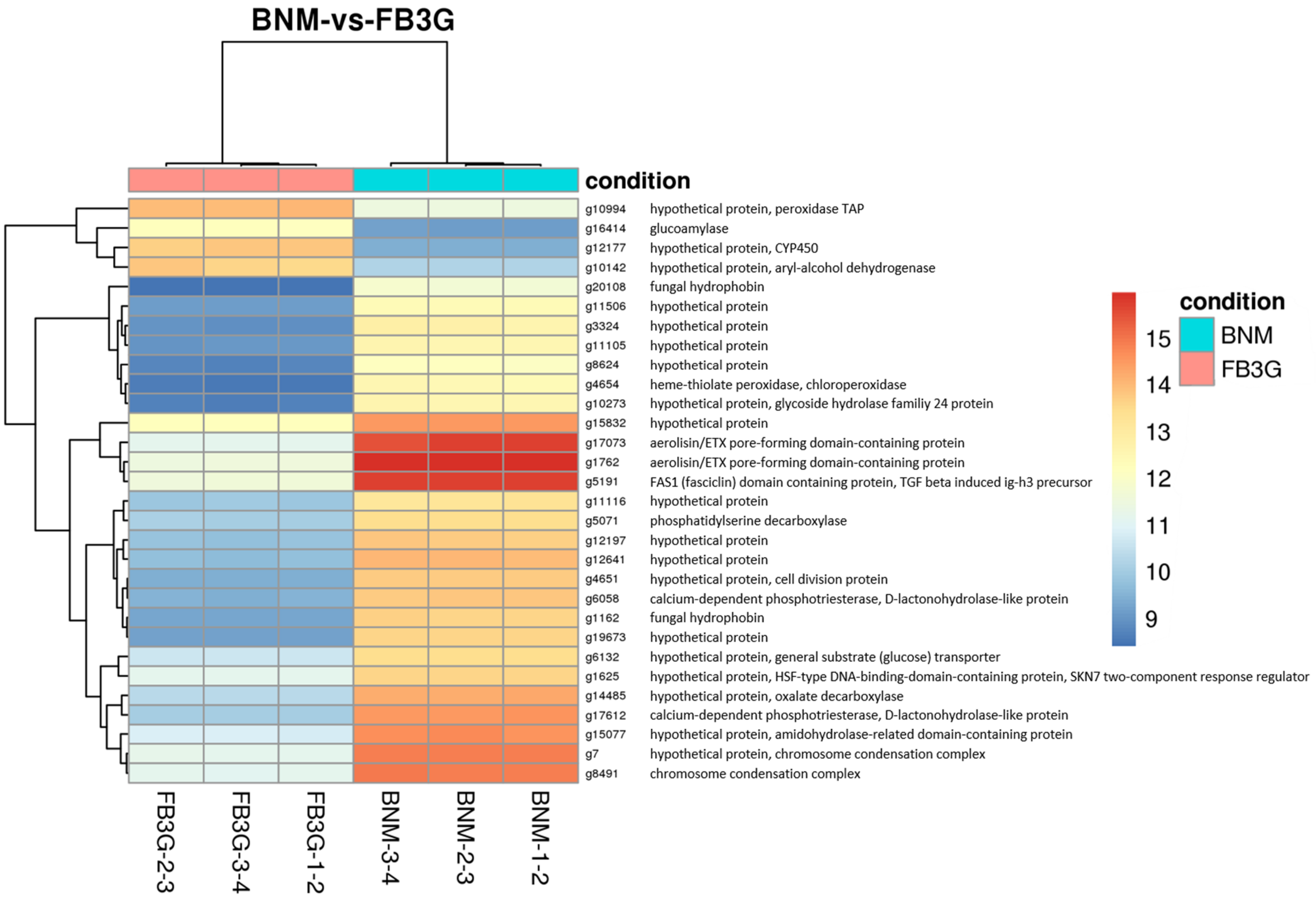

Figure S8. Bi-clustering of top 30 p_{adj}-value significant differentially expressed genes (I). Mycelium grown in BNM vs. FB3G medium was compared. Blast hits of respective genes have been added next to the gene ID.

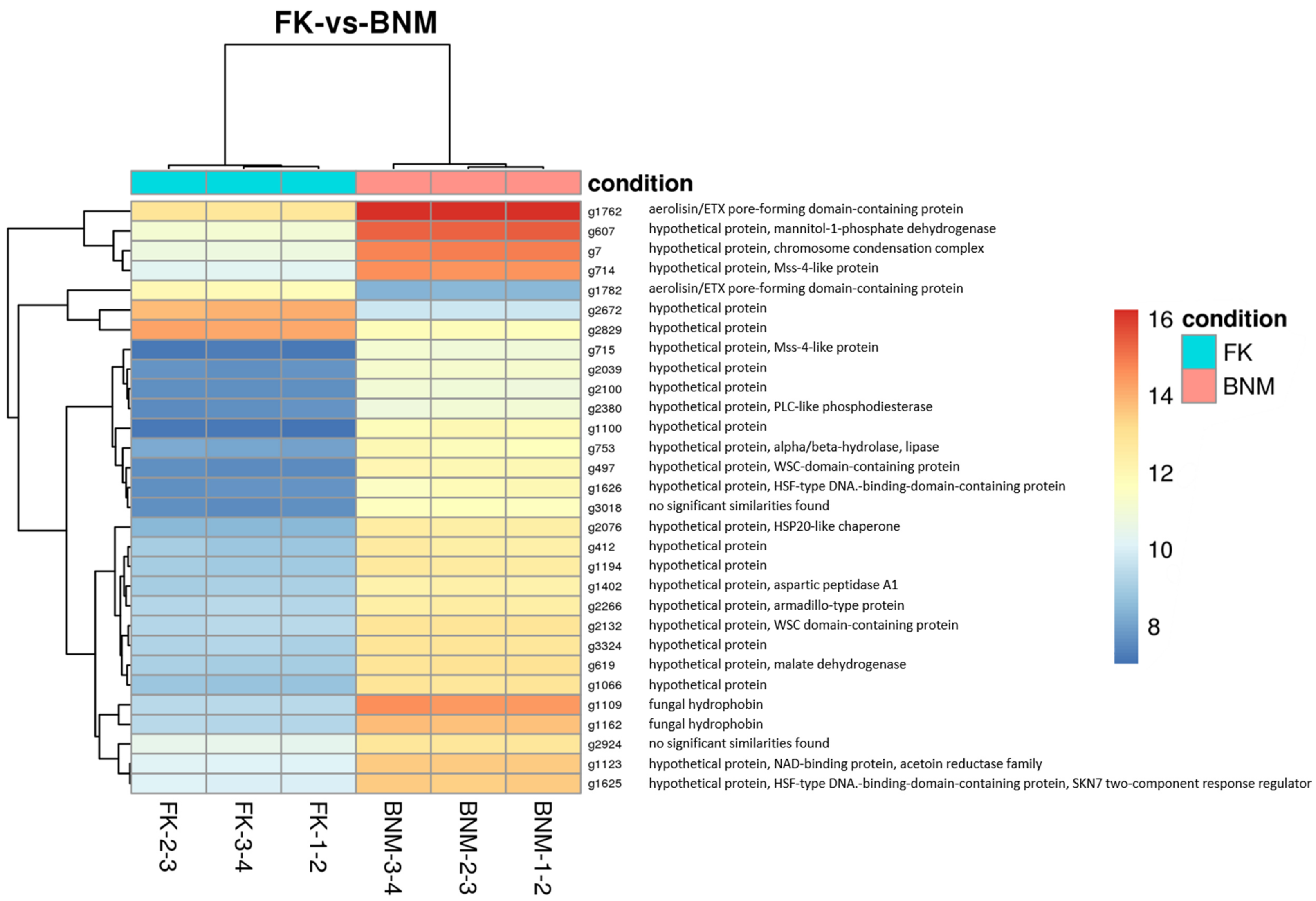

Figure S9. Bi-clustering of top 30 p_{adj}-value significant differentially expressed genes (II). Carpophores (FK) were compared with vegetative mycelium grown in BNM medium. Blast hits of respective genes have been added next to the gene ID.

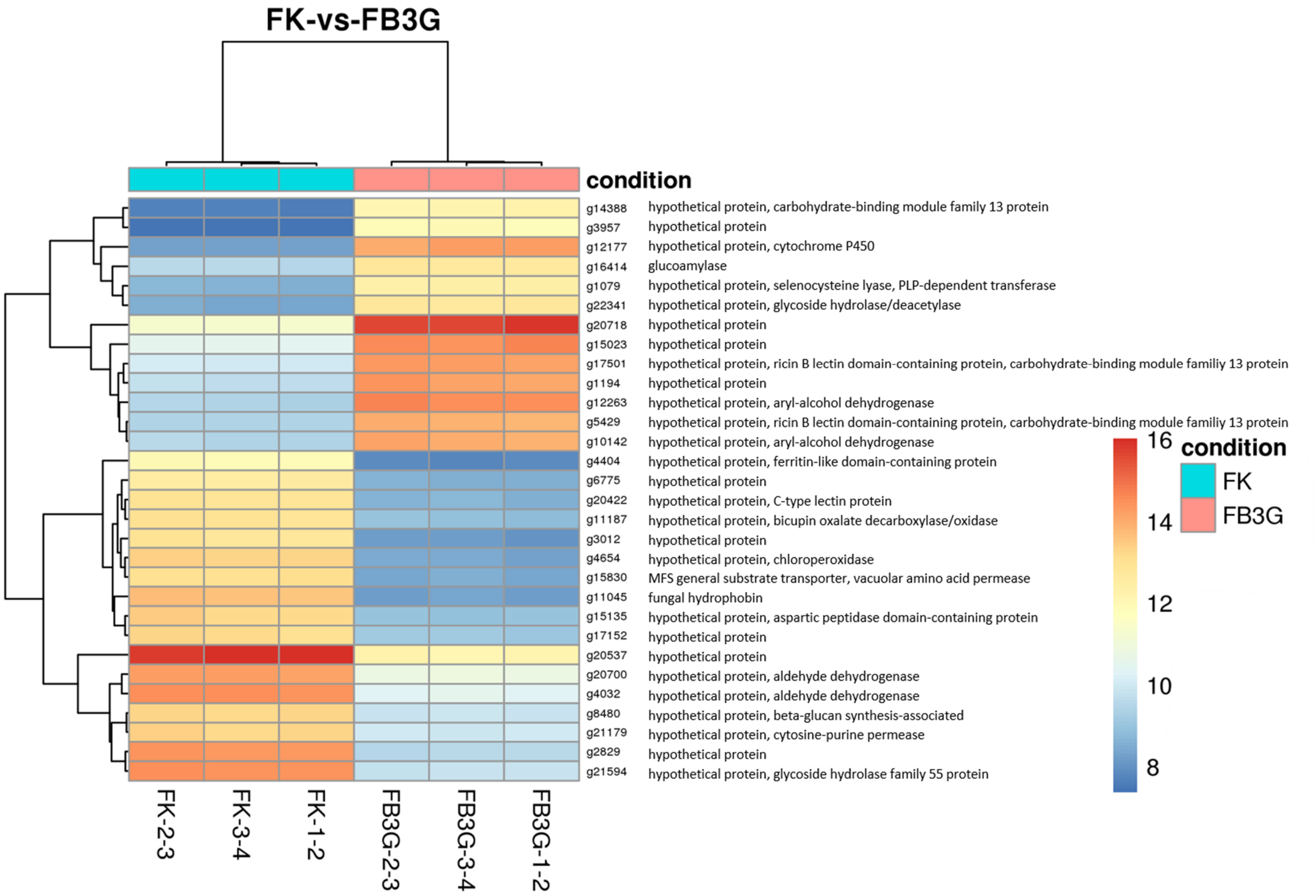

Figure S10. Bi-clustering of top 30 p_{adj}-value significant differentially expressed genes (III). Carpophores (FK) were compared with vegetative mycelium grown in FB3G medium. Blast hits of respective genes have been added next to the gene ID.

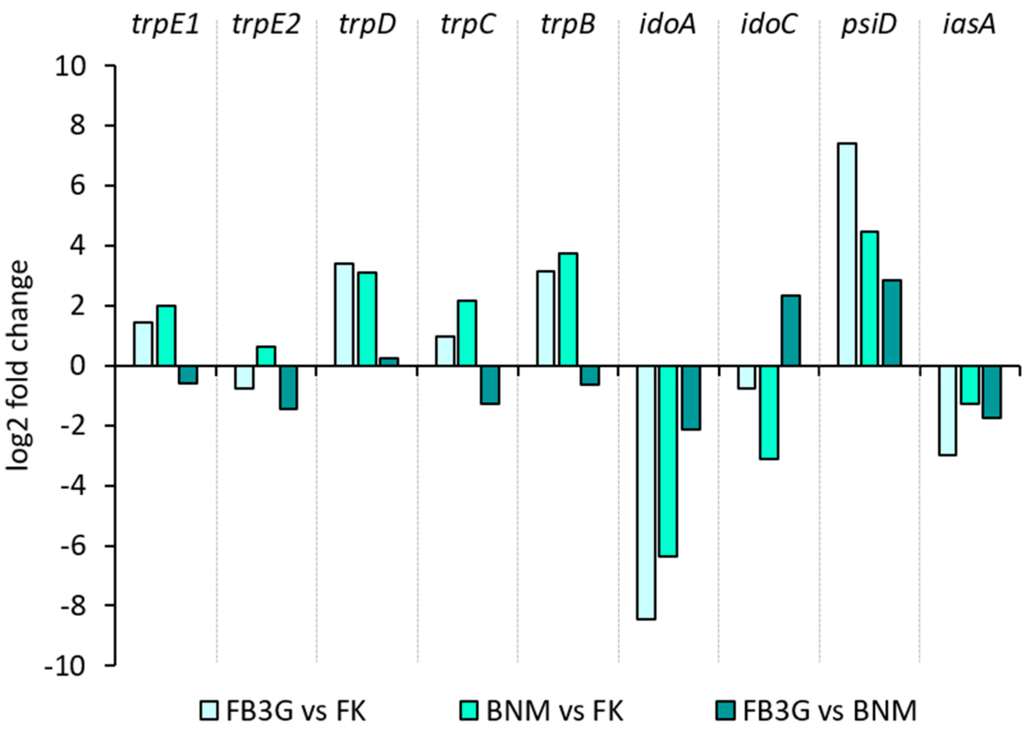

Figure S11. Differential expression of selected genes involved in the tryptophan metabolism in *P. mexicana*. Submerse-grown mycelia (in FB3G or BNM medium) as well as carpophores (FK) were used for RNA isolation and RNA-Seq. Genes that show positive $\log_2$-fold changes are upregulated in the respective second condition used for comparison.

Organism	Enzyme name	Enzyme type	Sequence	Accession#
Psilocybe mexicana	IasA	**AAS**	^{295}CTNFHKWGLVNFDCSTLWVRDRKLLTDALDITPP**F**LRT--KHGDAGTVIDYRNWHLGLGRRFR^{355}	PP316613
Psilocybe cubensis	PcDHPAAS	**AAS**	^{295}CTNFHKWGLVNFDCSALWVRDRKYLTDALDITPA**F**LRT--KQGDAGTVIDYRNWHLGLGRRFR^{355}	AYU58583
Arabidopsis thaliana	AtAAS	**AAS**	^{304}NMNAHKWFLTNFDCSLLWVKDQDSLTLALSTNPE**F**LKN--KASQANLVVDYKDWQIPLGRRFR^{364}	NP_849999
Olea europea	OeAAS	**AAS**	^{300}NMNAHKWFLTNFDCSALWIKDRSALIQSLSTNPE**F**LKN--KASEGNTVVDYKDWQIPLGRRFR^{360}	QJA07379
Petroselinum crispum	PcAAS	**AAS**	^{312}SLNAHKWFLTTLDCCCLWVRNPSALIKSLSTYPE**F**LKN--NASETNKVVDYKDWQIMLSRRFR^{372}	Q06086
Rosa hybrid cultivar	AAS	**AAS**	^{314}SFNPHKWLFTGMDCCCLWVKNPSVLASSLSTNPE**F**LRN--KASDSKQVVDYKDWQIALSRRFR^{374}	ABB04522
Arabidopsis thaliana	AtTYDC	**AAAD**	^{356}NMNAHKWLFANQTCSPLWVKDRYSLIDALKTNPE**Y**LEFKVKVSKKDTVVNYKDWQISLSRRFR^{418}	NP_001078461
Catharanthus roseus	CrTDC	**AAAD**	^{314}SLSPHKWLLAYLDCTCLWVKQPHLLLRALTTNPE**Y**LKN--KQSDLDKVVDFKNWQIATGRKFR^{374}	P17770
Papaver somniferum	TyDC9	**AAAD**	^{316}SLNAHKWFFTTLDCCCLWVKDSDSLVKALSTSAE**Y**LKN--KATESKQVIDYKDWQIALSRRFR^{376}	AAC61842
Oryza sativa	TDC	**AAAD**	^{325}SMSPHKWLMTCLDCTCLYVRDTHRLTGSLETNPE**Y**LKN--HASDSGEVTDLKDMQVGVGRRFR^{385}	AK069031
Thalictrum flavum	TYDC1	**AAAD**	^{313}SLNAHKWFFTTLDCCCLWVKEPSALIKALSTNPE**Y**LRN--KATESHQVVDYKDWQIALSRRFR^{373}	AAG60665

Figure S12. Sequence alignment of selected aromatic acetaldehyde synthases (AAS) and aromatic amino acid decarboxylases (AAAD). The phenylalanine residues (F) mediating aldehyde synthesis and the tyrosine residues (Y) conferring decarboxylase activity are highlighted in maroon and blue, respectively.

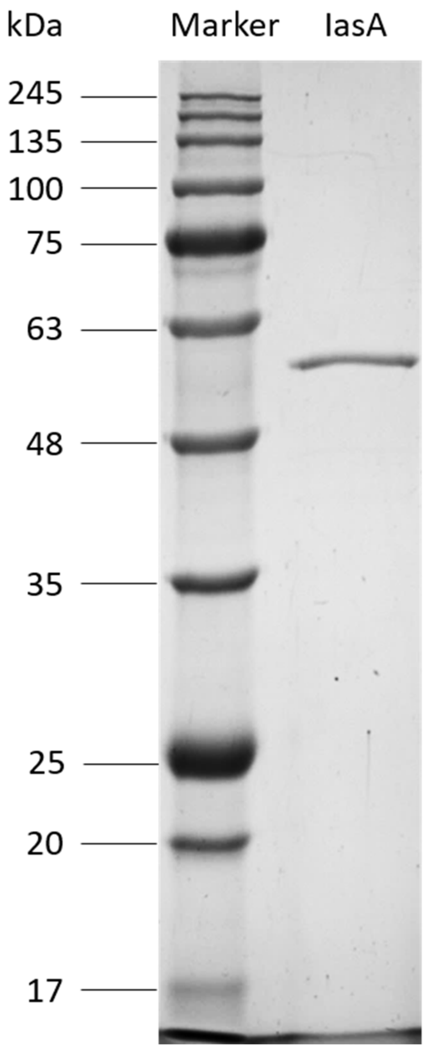

Figure S13. SDS-polyacrylamide gel electrophoresis of C-terminally hexahistidine-tagged *P. mexiana* IasA. The calculated protein mass for IasA-His$_6$ is 56.9 kDa. As protein standard (marker), the Blue Eye prestained marker (Jena Bioscience) was used.

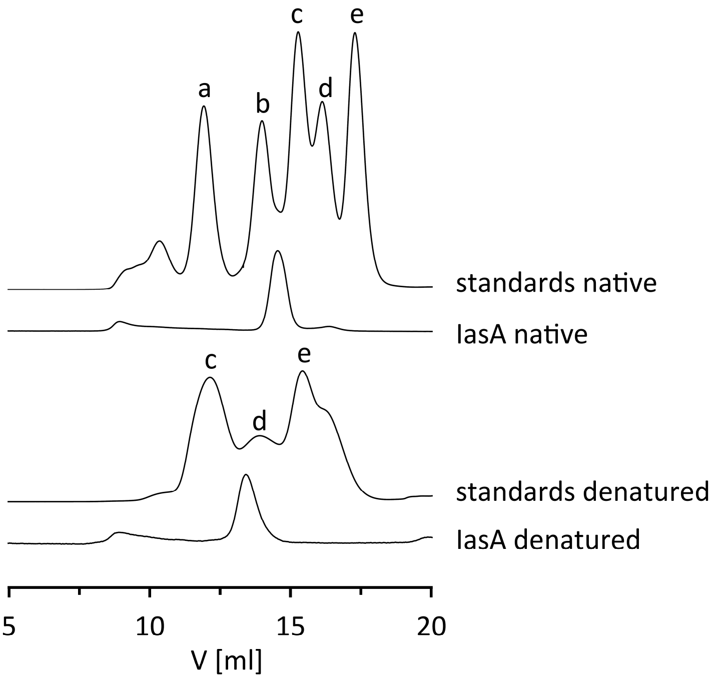

Figure S14. Size exclusion chromatography of native and denatured IasA. Chromatograms were extracted at λ = 280 nm (standards) and λ = 400 nm (IasA). Proteins were loaded onto a Superdex 200 column and eluted using 50 mM phosphate buffer with 150 mM NaCl (and 6 M urea to denature the proteins). As urea effects the migration, a calibration run was also performed under these conditions. Untreated IasA eluted at 14.5 ml (corresponding 113 kDa), the urea-treated IasA appeared at 13.4 ml (corresponding 52 kDa) indicating a homodimeric state in solution. The calculated mass of monomeric C-terminally His-tagged IasA is 56.9 kDa. Calibration tracks include ferritin (a, 440 kDa), aldolase (b, 158 kDa), conalbumin (c, 75 kDa), ovalbumin (d, 43 kDa), and carbonic anhydrase (e, 29 kDa).

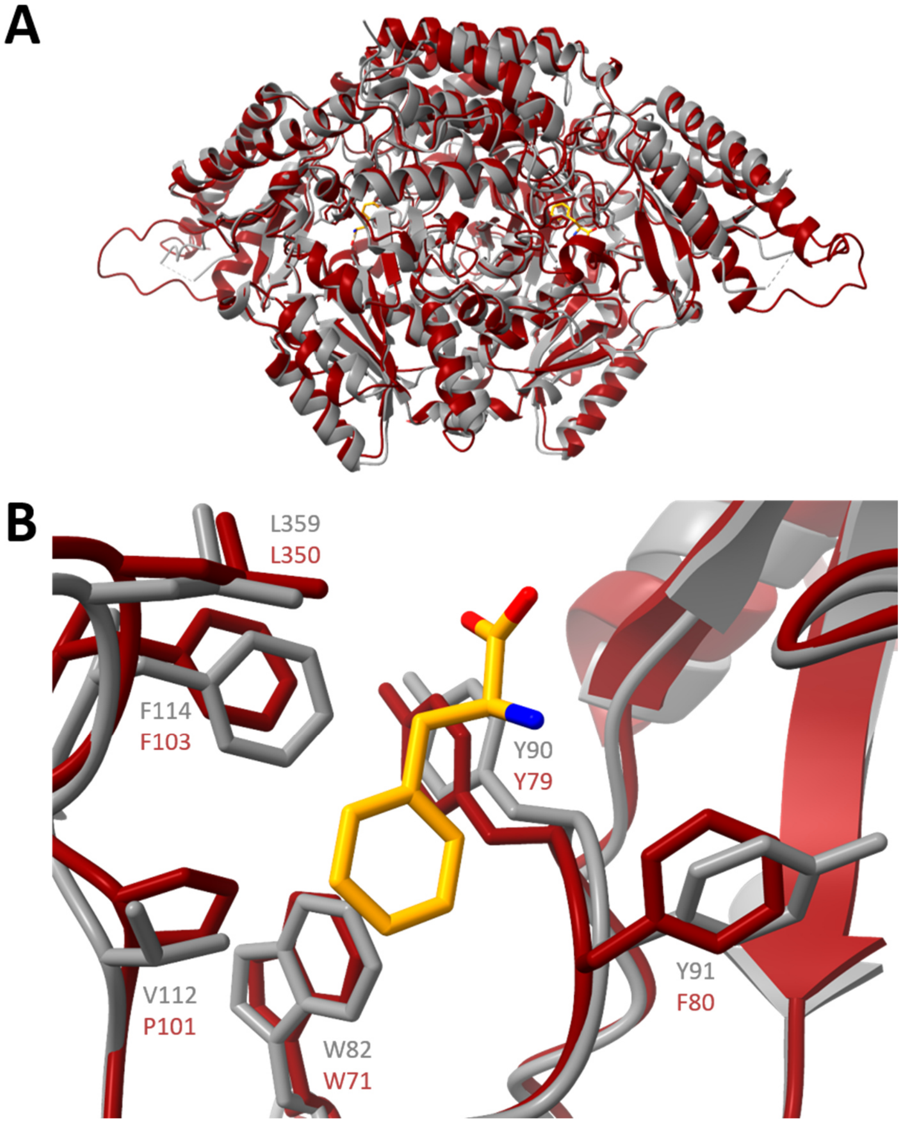

Figure S15. Structure prediction of *P. mexicana* IasA. The model of the *P. mexicana* IasA (maroon) was predicted by Alphafold [16]. ChimeraX software [17, 18] was used to superimpose the model with the experimentally verified structure of the *Arabidopsis thaliana* phenylacetaldehyde synthase (PDBe 6eei [19]; grey) co-crystallized with L-phenylalanine (orange). Panel A shows the *P. mexicana* IasA model superimposed twice, once onto chain A and at the same time onto chain B of the homodimeric *A. thaliana* phenylacetaldehyde synthase. Panel B shows close-up views of a portion of the enzymes around the active site. The structures of the respective amino acid residues are shown (Trp82, Tyr90, Tyr91, Val112, Phe114 and Leu359 of *A. thaliana* phenylacetaldehyde synthase and Trp71, Tyr79, Phe80, Pro101, Phe103 and Leu350 of *P. mexicana* IasA). The oxygen and nitrogen atoms of the ligand are marked in red and blue, respectively.

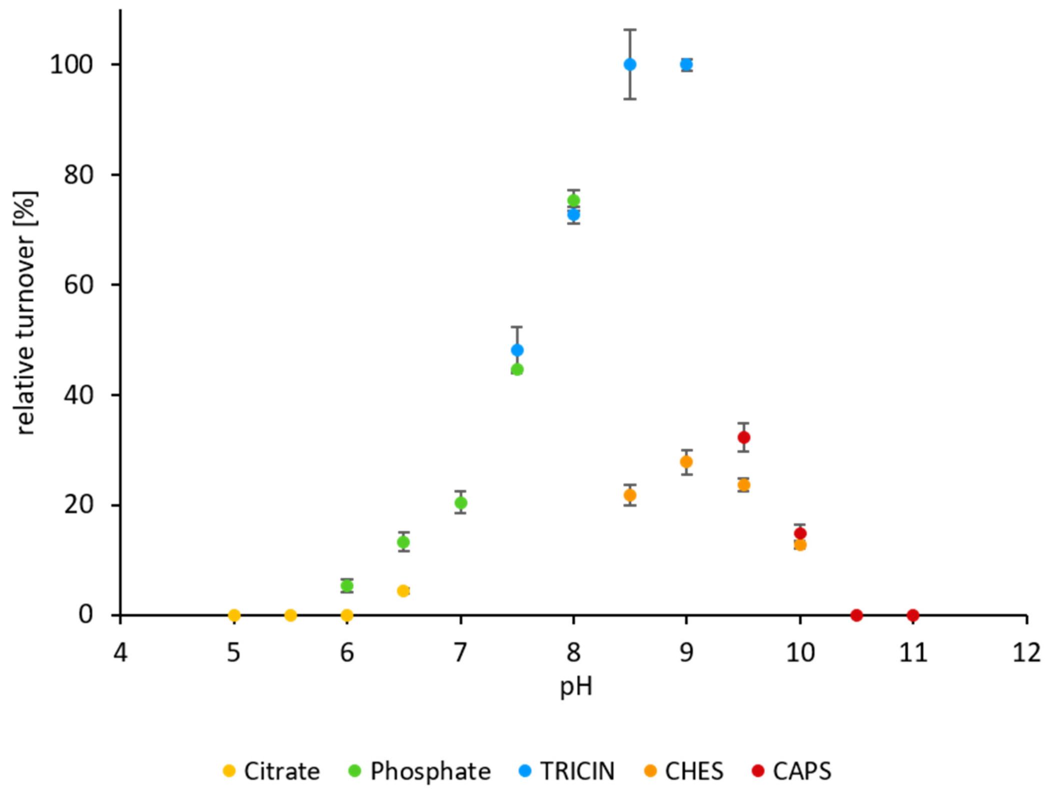

Figure S16. pH optimum of *P. mexicana* IasA. Error bars indicate the standard deviation (n=3).

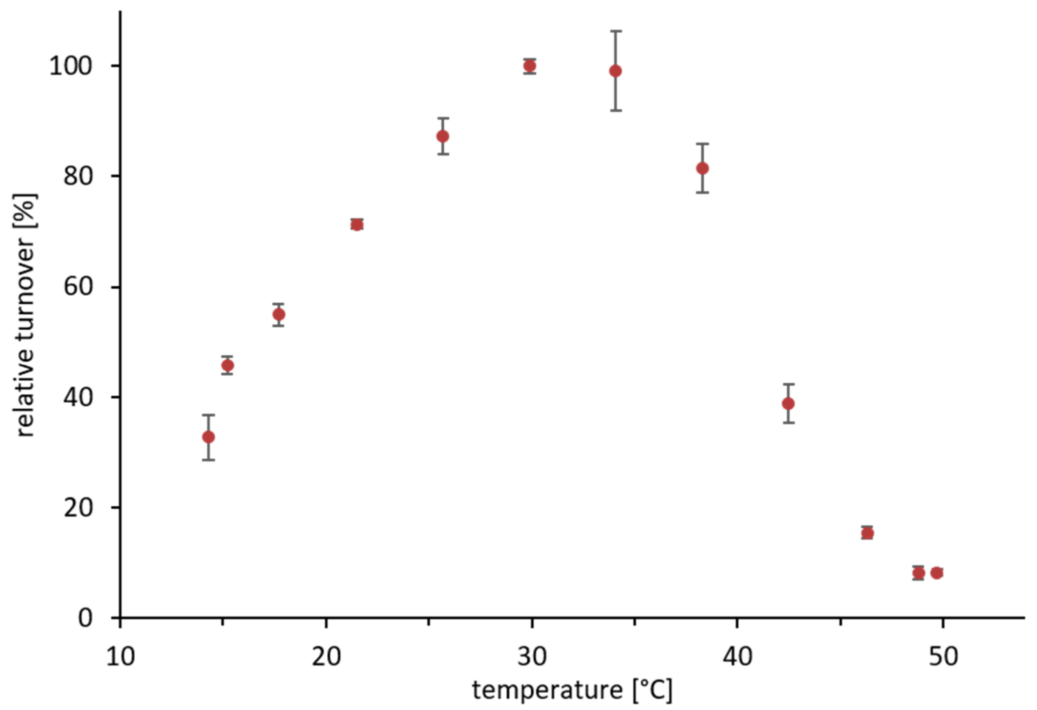

Figure S17. Temperature optimum of *P. mexicana* IasA. Error bars indicate the standard deviation (n=3).

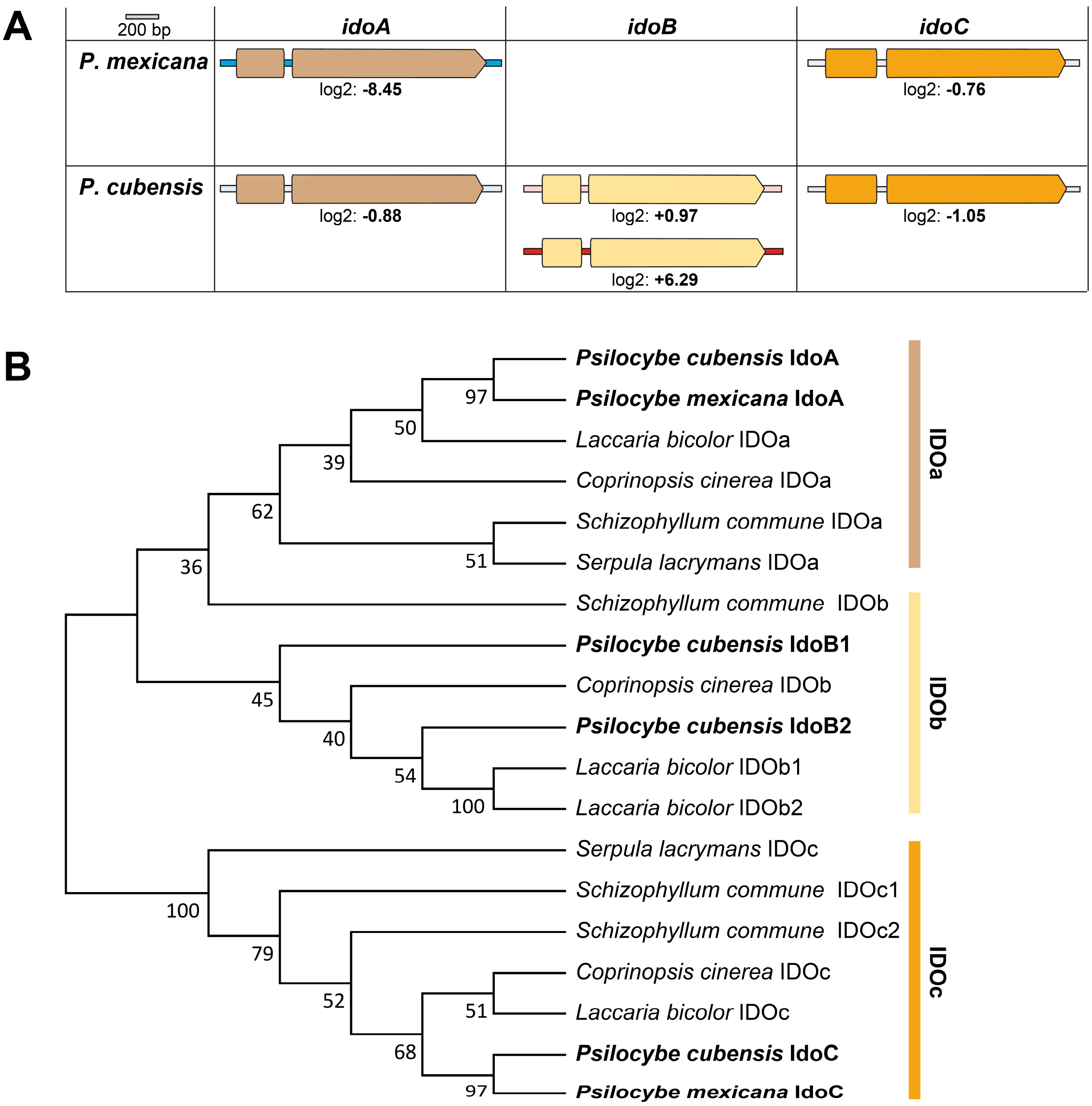

Figure S18. Analysis of indoleamine-2,3-dioxygenase genes and enzymes. (**A**) Indoleamine-2,3-dioxygenase genes with exon/intron architecture in *Psilocybe mexicana* and *Psilocybe cubensis*. Respective log_2-fold changes (mycelium grown in FB3G medium versus carpophores) are shown below. (**B**) Phylogenetic analysis of indoleamine-2,3-dioxygenases. The amino acid sequences were aligned using ClustalW2 [20] implemented in the MEGA X software [21]. The evolutionary history was inferred using the Maximum Likelihood method and Le_Gascuel_2008 model [22]. The bootstrap consensus tree inferred from 1000 replicates is taken to represent the evolutionary history of the analyzed taxa [23]. Branches corresponding to partitions reproduced in less than 50% bootstrap replicates are collapsed. The percentage of replicate trees in which the associated taxa clustered together in the bootstrap test (1000 replicates) are shown below the branches [23]. Initial tree(s) for the heuristic search were obtained automatically by applying Neighbor-Join and BioNJ algorithms to a matrix of pairwise distances estimated using the JTT model [24], and then selecting the topology with superior log likelihood value. A discrete Gamma distribution was used to model evolutionary rate differences among sites (5 categories (+G, parameter = 1.1238)). This analysis involved 19 amino acid sequences. All positions with less than 85% site coverage were eliminated, i.e., fewer than 15% alignment gaps, missing data, and ambiguous bases were allowed at any position (partial deletion option). There was a total of 107 positions in the final dataset. Evolutionary analyses were conducted in MEGA X [21]. The amino acid sequences of indolemanine-2,3-dioxygenases from *Coprinopsis cinerea*, *Laccaria bicolor*, *Schizophyllum commune* and *Serpula lacrymans* were taken from [25].

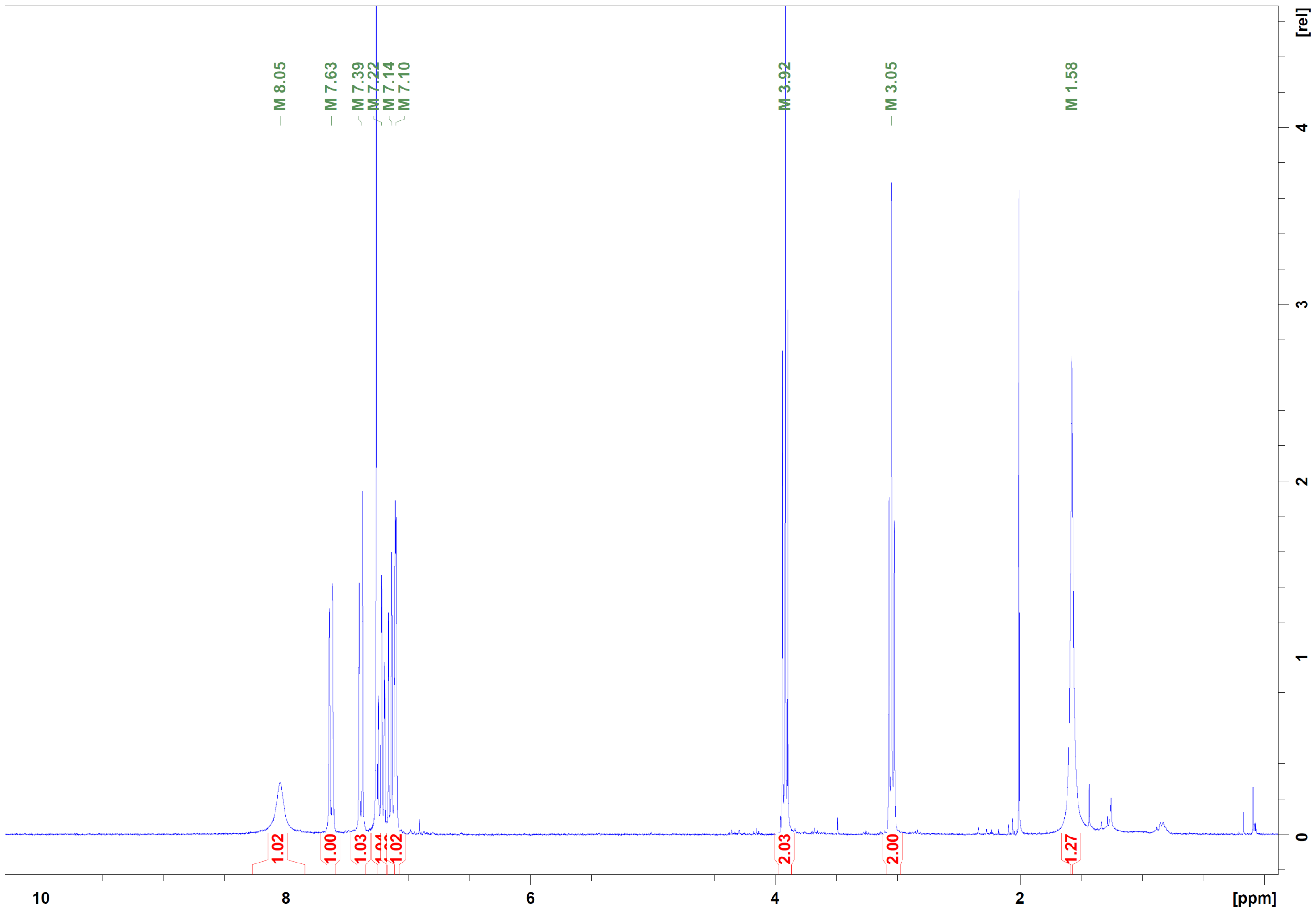

Figure S19. ^{1}H NMR spectrum of synthesized 2-(indol-3-yl)ethanol (tryptophol). 300 MHz, $CDCl_3$.

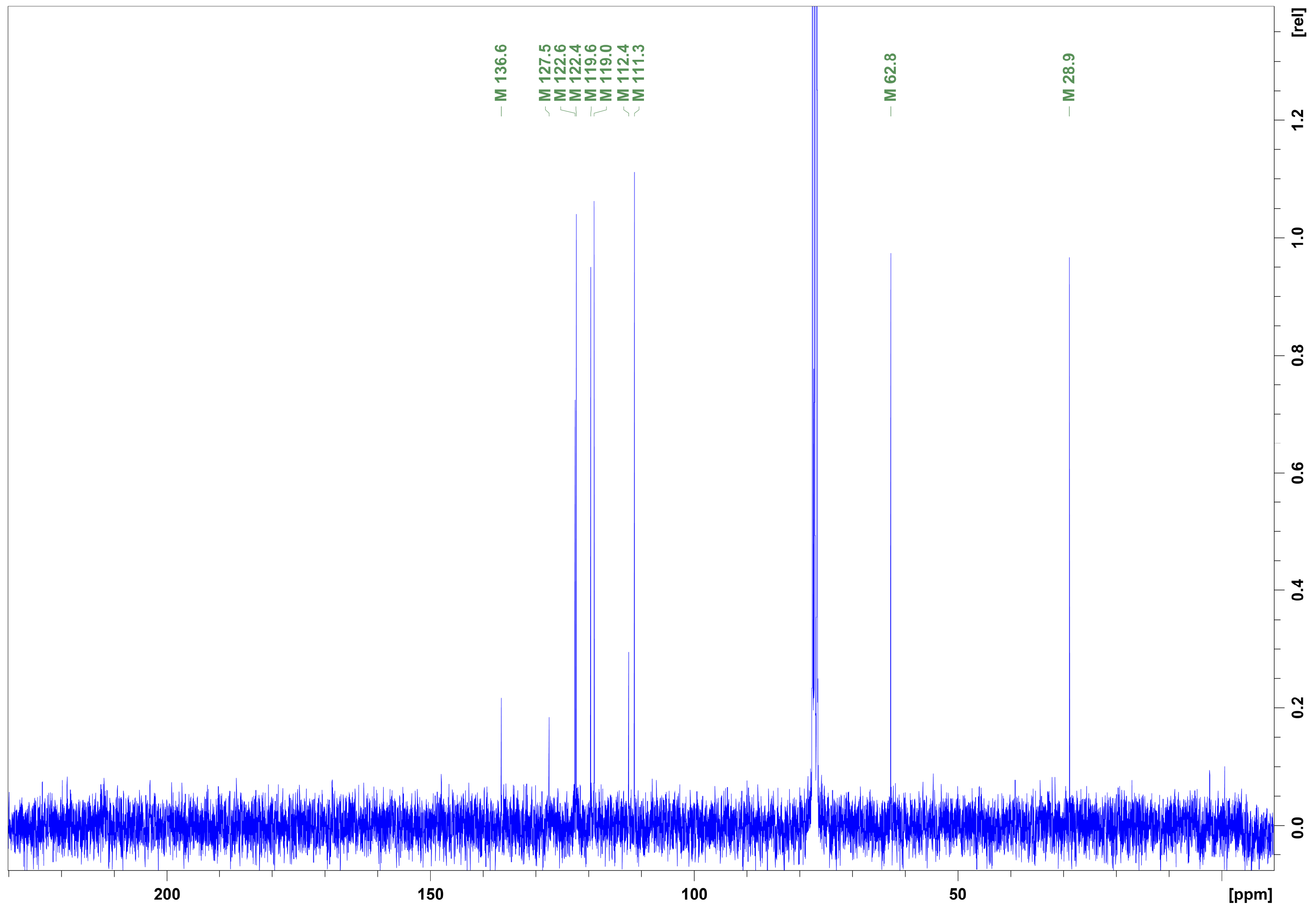

Figure S20. ^{13}C NMR spectrum of synthesized 2-(indol-3-yl)ethanol (tryptophol). 300 MHz, $CDCl_3$.

Sequence data 1. *P. mexicana* gene homologous to *Ganoderma lucidum gcn4*

Genomic sequence: A predicted intron is highlighted in red.

```
ATGCAGACAGTACACATCCCAAAGCAGGAGTATGATCAGTACGGGCCTTATACCACGCTTTTCGACATGGATGGG
GCGGAGCACTACGCCGACTTTGTCACCTGCCCTCCCACTACCACCCACATGAGGAACATTTCCTCAGAGTCTGGG
TCTACTTCTTCTTGTACGACAGTGGACGGTTCACTGCCGCTACCCACACACCCATCCATACAGGGCTTCAAACAG
AGGGCCGGAAGCCCTCAAGGATTCGTCTCTCTGGTTTCAGAGAGCCACGCCTCAACTCGTCGAATTCATCGACAC
AACCCCTCGGGGCCACAAGAGGATCACGCTCGCACAACCTTCCTACCCGAACCAAACGGGTCATCGTCGCATCCG
ACGATGAGGACAACTCCGAAGAGCACAACACACCGTTGCGCAAGGGCGCTACGGAGCAAGAGCGAATCGAGCACA
AACGTCGTCTCAATACGTTAGCTGCTAGACGCAGCCGCCGGCGTCGAGCCGACGAGCTCAAGGAGCTCCTCGAGA
CGGTCGATAGACTCGAGAAGGATTGTGTCAAATGGCGAACGCGCAGCGTGTTGCTACACGGGATGCTCGTCAAGA
ACGGATTCAACGCCCCTCCACTAAGTGACTGA
```

Translation of the respective coding sequence:

```
MQTVHIPKQEYDQYGPYTTLFDMDGAEHYADFVTCPPTTTHMRNISSESGSTSSWLQTEGRKPSRIRLSGFREPR
LNSSNSSTQPLGATRGSRSHNLPTRTKRVIVASDDEDNSEEHNTPLRKGATEQERIEHKRRLNTLAARRSRRRRA
DELKELLETVDRLEKDCVKWRTRSVLLHGMLVKNGFNAPPLSD
```

ClustalW alignment results:

22% pairwise identity/34% pairwise positive compared with GCN4 from *Ganoderma lucidum* (GenBank: QIX07536)

References

1. Du Y, Huang HY, Liu H, Ruan YP, Huang PQ: **Studies towards the Total Asymmetric Synthesis of the Pentacyclic Indole Alkaloid Arboflorine: Asymmetric Synthesis of a Key Intermediate**. *Synlett* 2011(4):565-568.
2. Torrens-Spence MP, Liu CT, Pluskal T, Chung YK, Weng JK: **Monoamine Biosynthesis via a Noncanonical Calcium-Activatable Aromatic Amino Acid Decarboxylase in Psilocybin Mushroom**. *ACS Chem Biol* 2018, **13**(12):3343-3353.
3. Blei F, Baldeweg F, Fricke J, Hoffmeister D: **Biocatalytic Production of Psilocybin and Derivatives in Tryptophan Synthase-Enhanced Reactions**. *Chemistry* 2018, **24**(40):10028-10031.
4. Dörner S, Rogge K, Fricke J, Schäfer T, Wurlitzer JM, Gressler M, Pham DNK, Manke DR, Chadeayne AR, Hoffmeister: **Genetic Survey of *Psilocybe* Natural Products**. *Chembiochem* 2022, **23**(14).
5. Veal D, Casselton LA: **Regulation of tryptophan metabolism in *Coprinus cinereus:* Isolation and characterisation of mutants resistant to 5-fluoroindole**. *Arch Microbiol* 1985, **142**:157-163.
6. Fernandes JD, Martho K, Tofik V, Vallim MA, Pascon RC: **The Role of Amino Acid Permeases and Tryptophan Biosynthesis in *Cryptococcus neoformans* Survival**. *Plos One* 2015, **10**(7):e0132369.
7. Choera T, Zelante T, Romani L, Keller NP: **A Multifaceted Role of Tryptophan Metabolism and Indoleamine 2,3-Dioxygenase Activity in *Aspergillus fumigatus*-Host Interactions**. *Front Immunol* 2017, **8**:1996.
8. Favilla LD, Herman TS, Goersch CDS, de Andrade RV, Felipe MSS, Bocca AL, Fernandes L: **Expanding the Toolbox for Functional Genomics in *Fonsecaea pedrosoi*: The Use of Split-Marker and Biolistic Transformation for Inactivation of Tryptophan Synthase (*trpB*) Gene**. *J Fungi (Basel)* 2023, **9**(2).
9. DeMoss JA, Wegman J: **An enzyme aggregate in the tryptophan pathway of *Neurospora crassa***. *Proc Natl Acad Sci U S A* 1965, **54**(1):241-247.
10. Miozzari G, Niederberger P, Hütter R: **Tryptophan biosynthesis in *Saccharomyces cerevisiae*: control of the flux through the pathway**. *J Bacteriol* 1978, **134**(1):48-59.
11. Yanofsky C, Platt T, Crawford IP, Nichols BP, Christie GE, Horowitz H, VanCleemput M, Wu AM: **The complete nucleotide sequence of the tryptophan operon of *Escherichia coli***. *Nucleic Acids Res* 1981, **9**(24):6647-6668.
12. Hütter R, Niederberger P, DeMoss JA: **Tryptophan biosynthetic genes in eukaryotic microorganisms**. *Annu Rev Microbiol* 1986, **40**:55-77.
13. Molina-Henares MA, Garcia-Salamanca A, Molina-Henares AJ, de la Torre J, Herrera MC, Ramos JL, Duque E: **Functional analysis of aromatic biosynthetic pathways in *Pseudomonas putida* KT2440**. *Microb Biotechnol* 2009, **2**(1):91-100.
14. Khan AR, Park GS, Asaf S, Hong SJ, Jung BK, Shin JH: **Complete genome analysis of *Serratia marcescens* RSC-14: A plant growth-promoting bacterium that alleviates cadmium stress in host plants**. *Plos One* 2017, **12**(2).
15. Gutiérrez-Preciado A, Yanofsky C, Merino E: **Comparison of tryptophan biosynthetic operon regulation in different Gram-positive bacterial species**. *Trends Genet* 2007, **23**(9):422-426.
16. Mirdita M, Schütze K, Moriwaki Y, Heo L, Ovchinnikov S, Steinegger M: **ColabFold: making protein folding accessible to all**. *Nat Methods* 2022, **19**(6):679-682.
17. Goddard TD, Huang CC, Meng EC, Pettersen EF, Couch GS, Morris JH, Ferrin TE: **UCSF ChimeraX: Meeting modern challenges in visualization and analysis**. *Protein Sci* 2018, **27**(1):14-25.
18. Pettersen EF, Goddard TD, Huang CC, Meng EC, Couch GS, Croll TI, Morris JH, Ferrin TE: **UCSF ChimeraX: Structure visualization for researchers, educators, and developers**. *Protein Sci* 2021, **30**(1):70-82.

19. Torrens-Spence MP, Chiang YC, Smith T, Vicent MA, Wang Y, Weng JK: **Structural basis for divergent and convergent evolution of catalytic machineries in plant aromatic amino acid decarboxylase proteins**. *Proc Natl Acad Sci U S A* 2020, **117**(20):10806-10817.
20. Larkin MA, Blackshields G, Brown NP, Chenna R, McGettigan PA, McWilliam H, Valentin F, Wallace IM, Wilm A, Lopez R *et al*: **Clustal W and Clustal X version 2.0**. *Bioinformatics* 2007, **23**(21):2947-2948.
21. Kumar S, Stecher G, Li M, Knyaz C, Tamura K: **MEGA X: Molecular Evolutionary Genetics Analysis across Computing Platforms**. *Mol Biol Evol* 2018, **35**(6):1547-1549.
22. Le SQ, Gascuel O: **An improved general amino acid replacement matrix**. *Mol Biol Evol* 2008, **25**(7):1307-1320.
23. Felsenstein J: **Confidence Limits on Phylogenies: An Approach Using the Bootstrap**. *Evolution* 1985, **39**(4):783-791.
24. Jones DT, Taylor WR, Thornton JM: **The rapid generation of mutation data matrices from protein sequences**. *Comput Appl Biosci* 1992, **8**(3):275-282.
25. Yuasa HJ, Ball HJ: **Indoleamine 2,3-dioxygenases with very low catalytic activity are well conserved across kingdoms: IDOs of Basidiomycota**. *Fungal Genet Biol* 2013, **56**:98-106.
